# Irradiation combined with PD-L1^−/−^ and autophagy inhibition enhances the antitumor effect of lung cancer via cGAS-STING-mediated T cell activation

**DOI:** 10.1016/j.isci.2022.104690

**Published:** 2022-06-30

**Authors:** Xinrui Zhao, Songling Hu, Liang Zeng, Xinglong Liu, Yimeng Song, Yuhong Zhang, Qianping Chen, Yang Bai, Jianghong Zhang, Haowen Zhang, Yan Pan, Chunlin Shao

**Affiliations:** 1 Institute of Radiation Medicine, Shanghai Medical College, Fudan University, Shanghai 200032, China; 2 State Key Laboratory of Radiation Medicine and Protection, School of Radiation Medicine and Protection, Medical College of Soochow University, Suzhou 215123, China

**Keywords:** microenvironment, radiation biology, immune response, cancer

## Abstract

Radiotherapy combined with immune checkpoint blockade has gradually revealed the superiority in the antitumor therapy; however, the contribution of host PD-L1 remains elusive. In this study, we found that the activation of CD8^+^ T cells was strikingly increased in both irradiated PD-L1-expressing primary tumor and distant non-irradiated syngeneic tumor in PD-L1-deficient mouse host, and thus enhanced radiation-induced antitumor abscopal effect (ATAE) by activating cGAS-STING pathway. Notably, the autophagy inhibitors distinctively promoted dsDNA aggregation in the cytoplasm and increased the release of cGAS-STING-regulated IFN-β from irradiated cells, which further activated bystander CD8^+^ T cells to release IFN-γ and contributed to ATAE. These findings revealed a signaling cascade loop that the cytokines released from irradiated tumor recruit CD8^+^ T cells that in turn act on the tumor cells with amplified immune responses in PD-L1-deficient host, indicating a potential sandwich therapy strategy of RT combined with PD-L1 blockage and autophagy inhibition.

## Highlights

• IR combined with PD-L1^−/−^ promotes the proliferation and differentiation of CD8^+^ T cells
• IFN-β from IR-tumor stimulates IFN-γ from bystander CD8^+^ T cells, more profound in PD-L1^−/−^ mice
• Autophagy inhibition boosts IR-induced dsDNA and cGAS-STING in the tumor of PD-L1^−/−^ mice
• Autophagy inhibition enhances IR-induced ATAE by activating cGAS-STING under PD-L1^−/−^ status

## Graphical abstract

**Figure:**
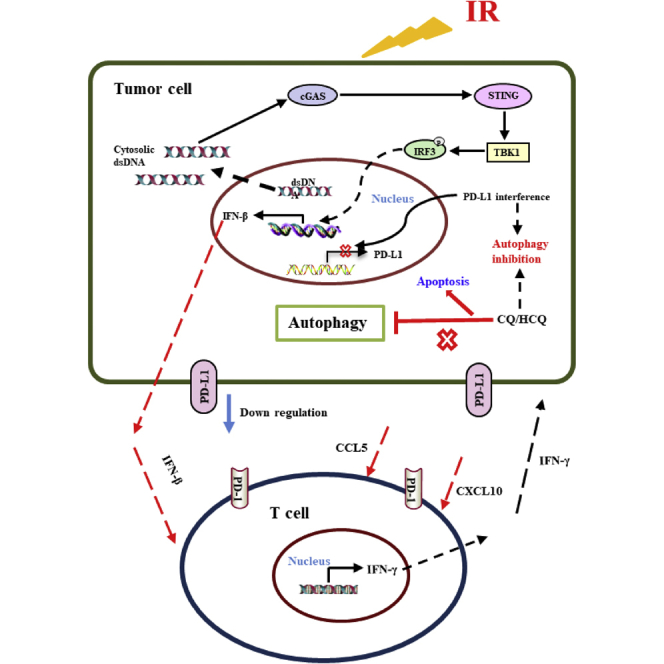

## Introduction

Radiotherapy (RT) is conventionally applied in antagonizing primary tumors and metastases. Radiation-induced shrinkage and regression of tumors result from a series of biological responses, including DNA damage, signal pathway conduction, modulation of tumor microenvironment, and inflammatory factors (Deng et al., 2014; Huang and Zhou, 2020; Mukherjee et al., 2014). Local tumor RT could also trigger the regression of metastases distant from irradiated field, which was termed as radiation-induced abscopal effect (Liu et al., 2018; Formenti and Demaria, 2009; Liauw et al., 2013). Increasing evidence showed that RT-induced tumoricidal effect could stimulate T cells to release interferon-1 (IFN-1) and create a pro-inflammatory milieu (Liu et al., 2018; Gulley et al., 2005). Radiation could also increase the accumulation of cytoplasmic double-strand DNA (dsDNA) (Pilones et al., 2015; Vanpouille-Box et al., 2018) that could be monitored by cyclic GMP-AMP synthase (cGAS), serving as a cytosolic DNA sensor. The spatially concentrated of cGAS could effectively promote the synthesis of second messenger cGAMP then bind to STING (stimulator of interferon genes) at endoplasmic reticulum (ER). Subsequently, STING functioned as a signal transduction platform to recruit and phosphorylate the downstream tank-binding kinase-1 (TBK1) and interferon regulator factor 3 (IRF3). The activated IRF3 undergone nuclear translocation and induced the transcription of type-I interferons (IFNs), particularly IFN-β (Kwon and Bakhoum, 2020; Kim et al., 2020; Li et al., 2020; Sen et al., 2019). Currently, the impact of immune responses including the implication of cGAS-STING signaling pathway in local RT-induced antitumor abscopal effect (ATAE) has become a high-profile issue (Zhao and Shao, 2020; Postow et al., 2012; Finazzi et al., 2018).

Immune checkpoints are critical inhibitory pathways to maintain self-tolerance and regulate the duration and amplitude of the physiological immune response of peripheral tissues to minimize collateral tissue damage (Zhang et al., 2017; Pardoll, 2012). Programmed cell death protein 1 (PD-1) and its ligand PD-L1 are undoubtedly one of the most noticeable immune checkpoint molecules. PD-1 (also known as CD279) is a type-I transmembrane receptor on the surface of T lymphocytes (Zhang et al., 2017; Freeman et al., 2000), and PD-L1 (also known as B7-H1 or CD274) is generally expressed on the resting T cells, B cells, dendritic cells, and macrophages. The binding of PD-1 and PD-L1 can restrict the hyper-activation of T cells to modulate the resolution of inflammatory response and sustain the ability of immunologic tolerance (Keir et al., 2006; Juneja et al., 2017). It is widely known that PD-L1 is often overexpressed on the surface of a variety of tumor cells, which impairs local immunity and further weakens the function of immune cells in preventing and attacking tumors (Shi et al., 2013). Therefore, targeting PD-1/PD-L1 axis has made a breakthrough progress in tumor immunotherapy (Brahmer et al., 2012; Topalian et al., 2012). However, emerging studies revealed that host immune system is essential for PD-L1 and PD-1 blockade therapy. PD-L1 in T cells, APCs, and host tissues, rather than cancer cell-intrinsic PD-L1, may play a critical role in suppressing antitumor immunity, as limiting T cell trafficking reduces the efficacy of blockade (Juneja et al., 2017; Tang et al., 2018; Lin et al., 2018). CD8^+^ T cell response was markedly enhanced and tumor growth was slower in the PD-L1 genetically deficient (PD-L1^−/−^) mice compared with PD-L1 wild-type (wt) mice (Tang et al., 2018; Latchman et al., 2004), indicating that PD-L1^−/−^ mice is an appropriate model for studying the mechanism of ATAE. Currently, RT combined with PD-1/PD-L1 blockade has achieved remarkable results in various tumor therapies (Park et al., 2015; Pfannenstiel et al., 2019; Chang et al., 2018). However, with respect to radioimmunotherapy, what kinds of immune cells and related cytokines playing major roles and how about the proportion of immune cells changed in the irradiated tumors, especially in the non-irradiated distal metastases, are still required to be deeply investigated.

Emerging studies have elucidated that autophagy inhibitor combined with PD-L1 blockade can expedite the death of cancer cells by encouraging apoptosis and disturbing the formation of vasculogenic mimicry (VM) (Ruan et al., 2019). How does the autophagy work in the immune system? Recent report demonstrated that hypoxia-induced autophagy enhanced the resistance of lung cancer cells to CD8^+^ T cell-mediated cell lysis (Noman et al., 2011) and facilitated breast cancer cells to evade NK-mediated cell killing through the degradation of NK-derived granzyme B (Baginska et al., 2013). Inhibition of autophagy by limiting the function of lysosome could activate the ability of CD8^+^ cytotoxic T lymphocytes (CTLs) to kill tumors (Khazen et al., 2016). Chloroquine (CQ) and hydroxychloroquine (HCQ) are the only drugs of autophagy inhibition that have been applied in clinical tumor therapy at present (Levy et al., 2017). In patients with glioblastoma treated concurrently with conventional surgery and radiochemotherapy, the addition of CQ prolonged the median survival (Sotelo et al., 2006; Briceño et al., 2007). For lung cancer, patients with breast cancer and ovarian cancer with brain metastases, CQ combined with radiotherapy also effectively increased the overall survival and well controlled the intracranial metastases (Eldredge et al., 2013; Rojas-Puentes et al., 2013). Therefore, autophagy inhibitors are available for improving the prognosis of patients with tumor after RT, where the immune responses may be involved.

In this study, we found that local irradiation (IR) on PD-L1-expressing lung carcinoma induced an antitumor effect on distal non-irradiated tumor, especially in PD-L1^−/−^ mouse host. Tumor RT in PD-L1^−/−^ mice triggered immune activation and promoted the recruitment of CD8^+^ T cells with IFN-γ secretion in both irradiated and abscopal lung cancer grafts. Meanwhile, autophagy inhibitor strengthened this ATAE by further enhancing the activation of CD8^+^ T cells, which was mediated by the cGAS-STING signaling pathway. Our findings indicate that the sandwich therapy of RT combined with PD-L1 blockage and autophagy inhibition may provide a new strategy for improving the curative effect of tumor therapy.

## Results

### PD-L1 deficiency enhanced radiation-induced ATAE

**Figure 1:**
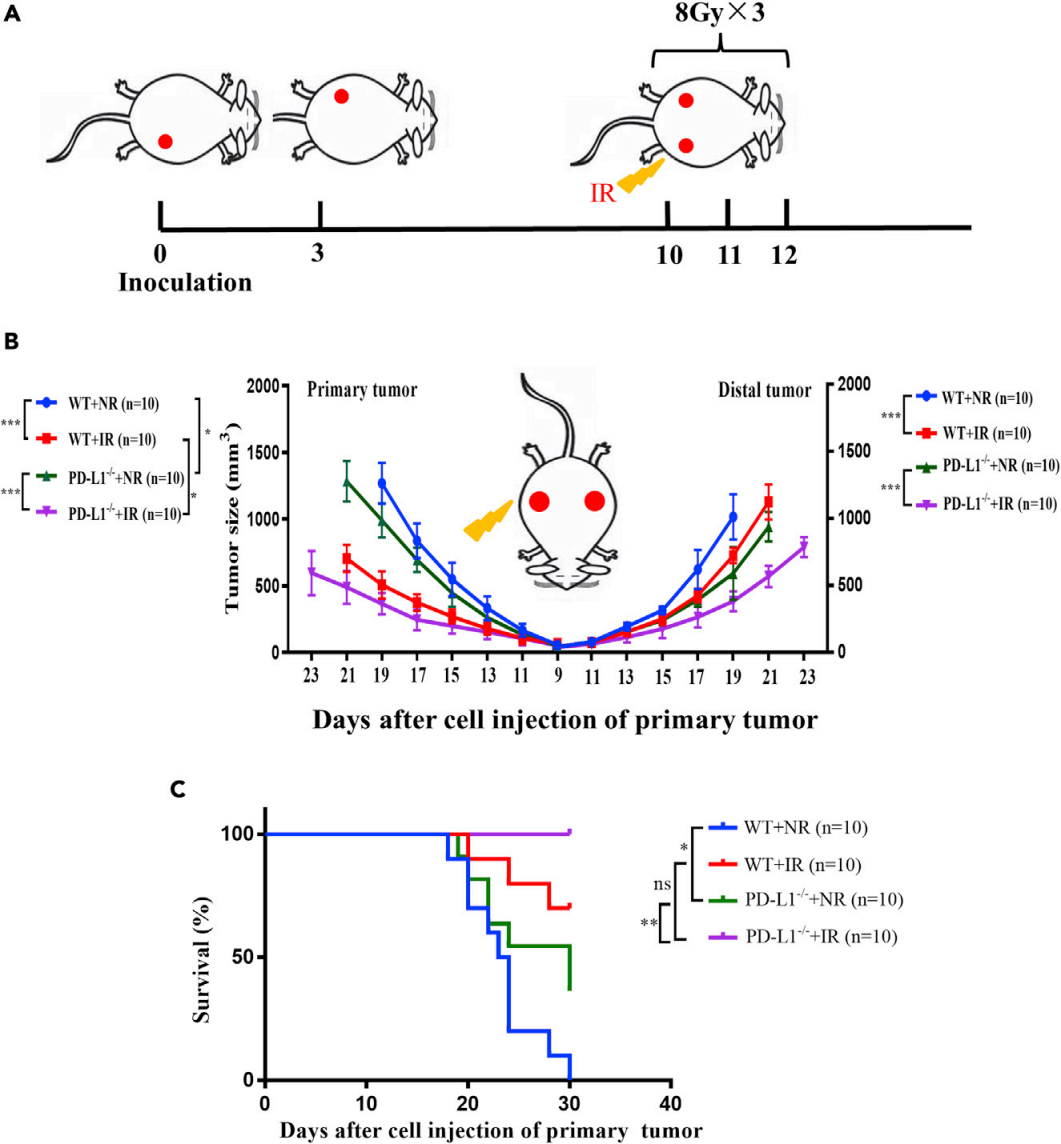
IR combined with PD-L1 deficiency enhanced the antitumor abscopal effect (ATAE) (A) Schema of the mice model for the investigation of radiation-induced ATAE. 2×10^6^ LLC cells were subcutaneously injected into the right flank on day 0 (as primary tumor) and contralateral left frank on day 3 (as distal tumor) of PD-L1 wt mice and PD-L1^−/−^ mice. On the 10th day after inoculation, the tumor in right flank was locally irradiated with 8 Gy × 3 fractions in 3 consecutive days. (B) Growth curves of primary and distal tumors in PD-L1 wt and PD-L1^−/−^ mice. Primary tumor was irradiated (IR) or non-irradiated (NR) (n = 10). Statistical analysis was performed by unpaired two-tailed Student’s *t* test, data were represented as means ± SEM. (C) The survival curves of PD-L1 wt and PD-L1^−/−^ mice bearing irradiated tumors (IR) or non-irradiated (NR) tumors. Log-rank Mantel–Cox test was applied for statistical analysis. ns, not statistically significant, ∗p < 0.05, ∗∗p < 0.01, ∗∗∗p < 0.001 between indicated groups. n = 10 mice each group.

To detect the synergistic effect of IR and PD-L1 deficiency in ATAE, we constructed a syngeneic tumor model in PD-L1 wt mice and PD-L1^−/−^ mice by subcutaneously inoculating Lewis lung carcinoma (LLC) cells into the right flank (as the primary tumor) and left flank (as the distal tumor) of a mouse. After growing to about 50 mm^3^, the primary tumor was irradiated with 24 Gy X-rays in 3 fractions in 3 consecutive days (8 Gy x 3) or non-irradiated (NR) (Figure 1A). In term of the tumor growth curves (Figure 1B), IR effectively retarded the growth of primary tumor in both wt and PD-L1^−/−^ mice. For the non-irradiated distal tumor, its growth rate was also reduced after primary tumor IR, which was much more evident in PD-L1^−/−^ mice. In addition, the growth rates of both primary and distal tumor in PD-L1 wt mice were faster than that in PD-L1^−/−^ mice. Moreover, the lifespan of tumor-bearing mice of PD-L1^−/−^ group was longer than that of PD-L1 wt mice, and RT on primary tumor prolonged the lifespan of both PD-L1 wt and PD-L1^−/−^ mice and even extended the lifespan of PD-L1^−/−^ mice to 30 days after tumor cells inoculation (Figure 1C). These results indicate that PD-L1 deficiency enhanced radiation-induced ATAE, benefited in tumor control, and hence increased the lifespan of tumor-bearing mice.

### PD-L1 deficiency enhanced IR-induced antitumor immunity

**Figure 2:**
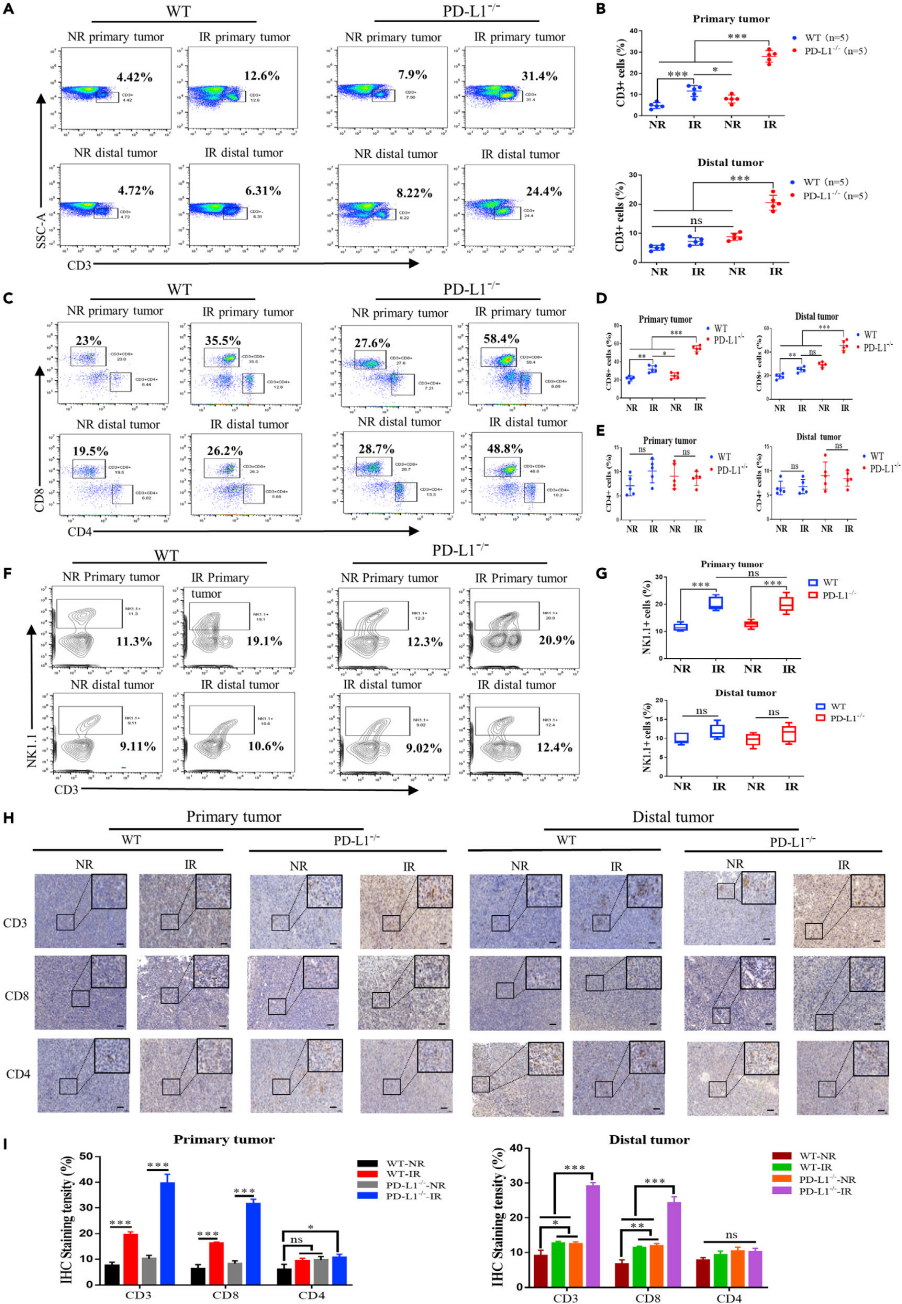
Analysis of IR-induced infiltration of intratumoral immune cells in PD-L1 wt and PD-L1 deficiency mice Primary tumor in PD-L1 wt and PD-L1^−/−^ mice was irradiated (IR) or non-irradiated (NR). Tumors were collected at day 21 after LLC cells injection. (A and B) Flow cytometric analysis of the ratio of infiltrated CD3^+^ T cells in primary and distal tumors in PD-L1 wt and PD-L1^−/−^ mice. Primary tumor was irradiated (IR) or non-irradiated (NR). (C–E) The percentages of CD4^+^ and CD8^+^ T cells within CD3^+^ T cell population in primary and distal tumor were detected by flow cytometry. (F and G) The percentage of NK cells of primary and distal tumors was detected by flow cytometry. (H) The representative images of IHC staining of CD3^+^, CD4^+^, and CD8^+^ T cells in primary and distal tumor tissues. Scale bars, 50 μm. All data represent at least three independent experiments. (I) The percentage of CD3, CD4, and CD8 positive cells in primary and distal tumor tissues according to above IHC staining. ns, not statistically significant, ∗p < 0.05, ∗∗p < 0.01, ∗∗∗p < 0.001 between indicated groups. n = 5 mice each group.

Considering the pivotal value of PD-1/PD-L1 axis in tumor immunity (Sun et al., 2018), we wondered how IR combined PD-L1^−/−^ triggered the immune response to achieve an antitumor efficacy and thus profiled the composition of tumor-infiltrating T lymphocytes after IR on the primary tumor. As shown in Figures 2A and 2B, the quantity of infiltrated CD3^+^ T cells in the irradiated primary tumor was significantly increased at day 10 after IR (8 Gy × 3) in PD-L1 wt mice, and it was more strikingly elevated in PD-L1^−/−^ mice. Meanwhile, after primary tumor IR, the ratio of infiltrated CD3^+^ T cells in the distal tumor of PD-L1 wt mice had no significantly alteration in comparison with non-IR group, but it was also strikingly increased in PD-L1^−/−^ mice. Then, we investigated the subset of T cells exerting the main function of antitumor and found that the percentage of CD3^+^CD8^+^ cells in the irradiated primary tumor was increased to 1.5-fold and 2.7-fold of non-irradiated tumor in PD-L1 wt and PD-L1^−/−^ mice, respectively (ures 2C–2E). This IR also significantly increased the ratios of CD3^+^CD8^+^ cells in the distal tumors of both PD-L1 wt and PD-L1^−/−^ mice. However, no significant difference in CD4^+^ cell population was observed between above groups before and after IR.

IR-induced alteration of another tumor killing cells NK cells was also examined. It was found that the ratio of NK cells in the primary tumor of IR group was obviously higher than that of NR group, but the extent of this increase was independent of PD-L1 status (Figures 2F and 2G). For the distal tumor, the primary tumor IR did not change the number of infiltrated NK cells whether in PD-L1 wt or PD-L1^−/−^ mice. Moreover, the examination results of immunohistochemical (IHC) staining of CD3^+^, CD4^+^, and CD8^+^ T cells in the tumor tissues were consistent with those of above flow cytometry analyses (Figures 2H and 2I). Taken together, tumor IR effectively promoted the recruitment of CD3^+^ T lymphocytes, especially the infiltration of CD8^+^ T cells, into both irradiated primary tumor and non-irradiated distal tumors, and this recruitment response was much stronger in PD-L1^−/−^ mice in comparison with PD-L1 wt mice. Therefore, CD3^+^CD8^+^ T cells, rather than CD3^+^CD4^+^ T cells, may exert the prominent antitumor immunity role in radiotherapy.

### IR combined PD-L1 deficiency rescued tumor-induced splenomegaly

**Figure 3:**
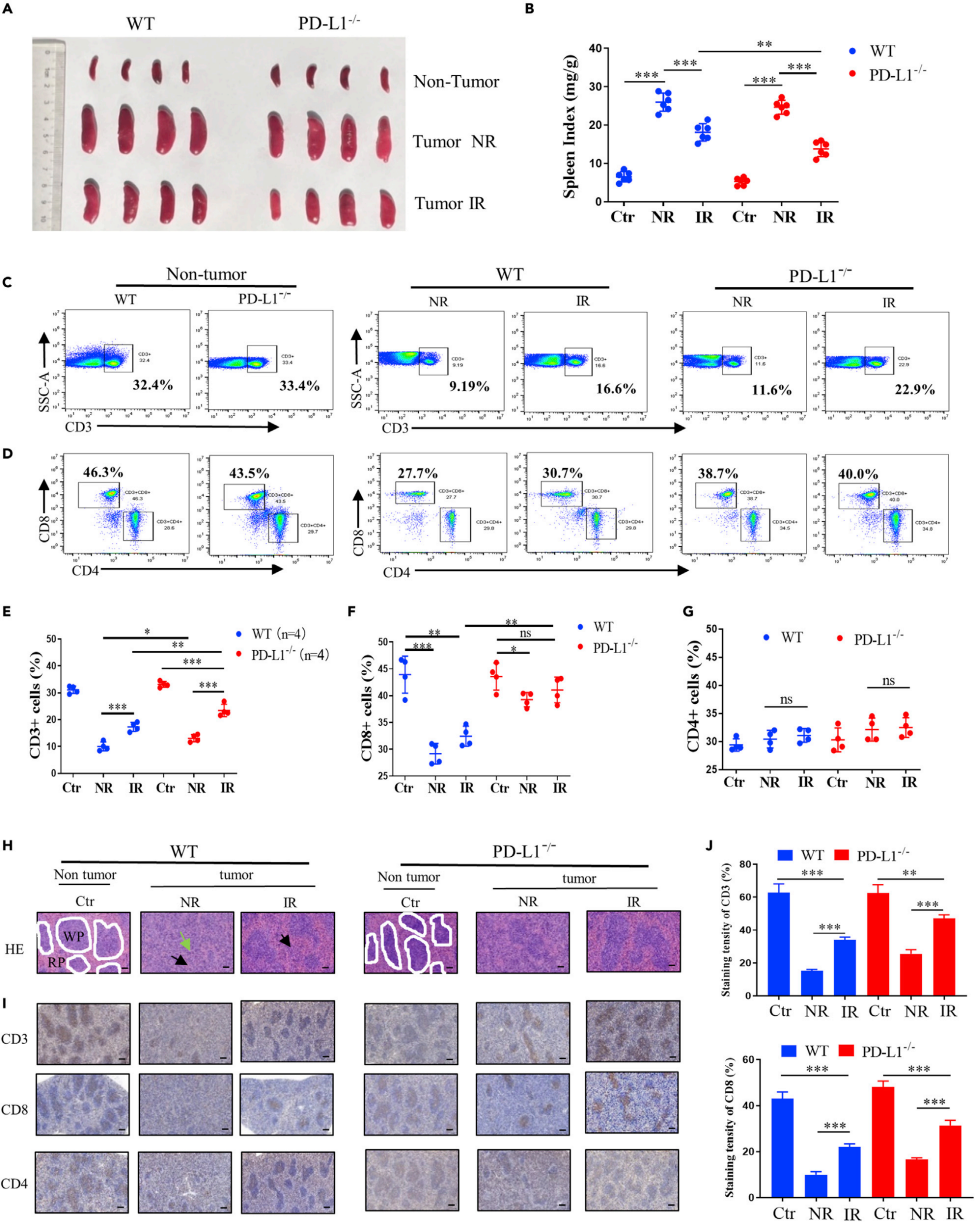
IR plus PD-L1 deficiency effectively relieved splenomegaly caused by tumor growth Primary tumor in W PD-L1 wt T and PD-L1^−/−^ mice was irradiated (IR) or non-irradiated (NR). Spleens were collected at day 21 after LLC cells injection. (A and B) Representative images of the spleens and the spleen ind. ex of PD-L1 wt and PD-L1^−/−^ mice bearing tumor irradiated (IR) or non-irradiated (NR). (C and E) Flow cytometric analysis of the ratio of infiltrated CD3^+^ T cells in spleen. (D, F, and G) Flow cytometric analysis of the ratios of CD4^+^ and CD8^+^ T cells within CD3^+^ T cells in spleen. (H) HE-staining images of spleen tissues from above indicated mice. White pulp (WP) in the normal spleen is outlined with white line. Damaged white pulp was indicated by green arrow, and extra-medullary hematopoietic foci and mature neutrophil in red pulp (RP) are indicated by black arrow. Scale bars, 100 μm. (I and J) IHC staining of CD3, CD4, and CD8 cells in spleen tissues from above indicated mice (I) and the corresponding quantitative analyses (J). Scale bars, 200 μm. ns, not statistically significant, ∗p < 0.05, ∗∗p < 0.01, ∗∗∗p < 0.001 between indicated groups. n = 4 mice each group.

As a main immune organ, the mice spleens were obviously enlarged along with the rapid growth of tumors both in PD-L1 wt and PD-L1^−/−^ mice. But the local tumor IR effectively restrained the abnormal enlargement of spleens, especially in tumor-bearing PD-L1^−/−^ mice (Figures 3A and 3B). Flow cytometry analysis of the immune cells in the spleens of tumor-bearing mice showed that the proportion of CD3^+^ T cells in the IR group was higher than that in the NR group, and more CD3^+^ T cells were detected in the spleens of PD-L1^−/−^ mice, although the proportions of intrasplenic CD3^+^ cells in IR groups were still less than that of non-tumor control group (Figures 3C and 3E). Meanwhile, the ratios of CD3^+^CD8^+^ cells in the spleens of tumor-bearing mice were slightly recovered by IR in both PD-L1 wt and PD-L1^−/−^ groups (Figures 3D and 3F). However, there was no significant difference of the proportion of CD3^+^CD4^+^ cells between each group (Figures 3D and 3G). Hematoxylin-eosin (HE) staining of the spleen also revealed that in the non-tumor PD-L1 wt and PD-L1^−/−^ mice, no obvious abnormalities were observed in the structure of white pulp (WP) and red pulp (RP) of spleens. While in the spleens of the tumor-bearing PD-L1 wt and PD-L1^−/−^ group, the WP was severely damaged, as shown by the dramatically decreased number of WP and the smaller remaining volume (green arrow). Meanwhile, massive extramedullary hematopoietic foci and more mature neutrophil infiltration were detected in the red pulp (black arrow), which may lead to splenomegaly. However, IR significantly decreased the damage of spleens in tumor-bearing PD-L1 wt mice; moreover, the relieved damage in the spleen by IR was further improved in the tumor-bearing PD-L1^−/−^ mice (Figure 3H). The analysis results of IHC staining of CD3^+^, CD4^+^, and CD8^+^ T cells on the splenocytes were consistent with those of flow cytometry assay (Figures 3I and 3J). In general, our results demonstrated that IR effectively mitigated the white pulp damage in spleens and alleviated the abnormal spleen enlargement in tumor-bearing mice, especially under the situation of PD-L1 deficiency.

### IR-combined PD-L1 deficiency activated CD8^+^ T cells

Studies had demonstrated that PD-L1^−/−^ CD8^+^ T cells exhibit an enhanced cytotoxic phenotype (Diskin et al., 2020). We also detected the basal level of immune cell and CD8^+^ T cell function in the spleen of PD-L1 wt and PD-L1^−/−^ mice (Figure S1) and found that there was no significant difference in the proportion of CD3^+^ T, CD4^+^ T, and CD8^+^ T cells in splenocytes (Figures S1A and S1B). Similarly, there was no obvious difference in the proliferative capacity of CD8^+^ T cells between these two groups (Figure S1C). But notably, the ratio of memory-like (CD44^hi^CD62L^low^) CD8^+^ T cells (Figure S1D), PD-1 expression in CD8^+^ T cells (Figure S1E), and IFN-γ released from CD8^+^ T cells (Figure S1F) was much more significantly increased in PD-L1^−/−^ mice than in PD-L1 wt mice.

**Figure 4:**
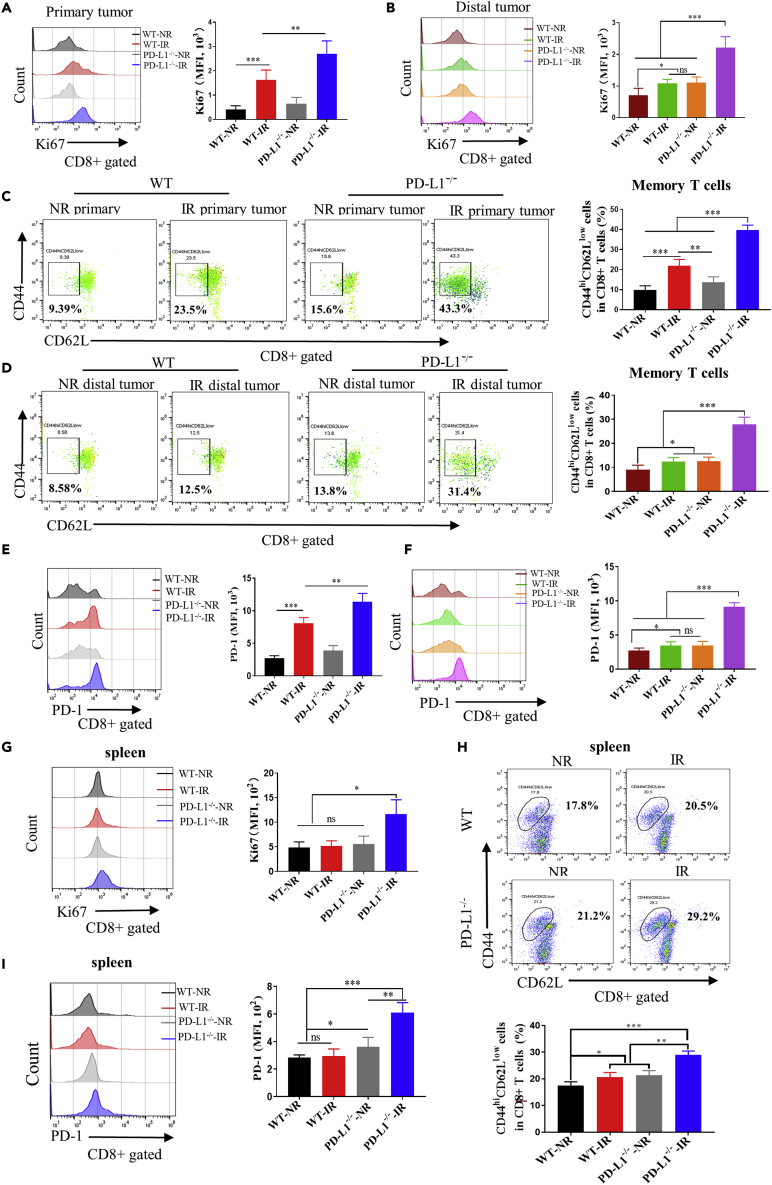
IR-combined PD-L1 deficiency activated the function of CD8^+^ T cells for antitumor immunity Primary tumor in PD-L1 wt and PD-L1^−/−^ mice was irradiated (IR) or non-irradiated (NR). (A and B) Under the gating of CD8^+^ T cells, the Ki67 indicator of primary tumor (A) and distal tumor (B) was detected by flow cytometry, and the mean fluorescence intensity (MFI) value of each group was analyzed and represented in histogram. (C and D) Flow cytometric analysis of the ratio of memory-like (CD44^hi^CD62L^low^) CD8^+^ T cells in primary and distal tumors of PD-L1 wt and PD-L1^−/−^ mice. (E and F) Flow cytometric analysis of PD-1 expression in the CD8+T cells infiltrated in the primary tumors (E) and distal tumors (F), and the MFI value of each group was represented in histogram. (G) Flow cytometric analysis of the Ki67-positive cells in CD8^+^ T cells in spleen. (H) Flow cytometric analysis of the percentage of memory-like cells displaying CD44^hi^CD62L^low^ in CD8^+^ T cells in spleen. (I) Flow cytometric analysis of PD-1 expression in CD8^+^ T cell in spleen. ns, not statistically significant, ∗p < 0.05, ∗∗p < 0.01, ∗∗∗p < 0.001 between indicated groups. n = 5 mice each group (A–I) and n = 4 mice each group (G-I).

We then examined the changes of CD8^+^ T cell function after tumor IR of in PD-L1 wt and PD-L1^−/−^ mice. It was found that the proportion of Ki67+CD8^+^ T cells increased in both primary and distal tumor after IR treatment, especially in the tumor of PD-L1^−/−^ mice (Figures 4A and 4B), indicating that the proliferation ability of intratumoral CD8^+^ T cells could be promoted by IR, which was further enhanced under the situation of PD-L1 deficiency. Moreover, the activity of CD8+ T cells, i.e., the proportion of CD44^hi^CD62L^low^ CD8^+^ memory-like T cells in primary tumor was increased after IR and it was more profound in PD-L1^−/−^ mice (Figure 4C). While in the distal tumor, the memory-like T cells only significantly aggregated in PD-L1^−/−^ mice after IR (Figure 4D). In addition, the expression of PD-1 in CD8+T cells of irradiated primary tumor was increased in PD-L1 wt mice and more significantly elevated in PD-L1^−/−^ mice (Figure 4E), but the expression of PD-1 was only obviously increased in the distal tumor of irradiated PD-L1^−/−^ mice (Figure 4F). Moreover, the proportions of Ki67+ CD8^+^ T cells and CD44^hi^CD62L^low^ CD8^+^ memory-like T cells in the spleen of tumor-irradiated PD-L1^−/−^ mice were significantly higher than that in PD-L1 wt mice and non-irradiated PD-L1^−/−^ mice (Figures 4G and 4H), and the expression of PD-1 in the intrasplenic CD8^+^ T cells of tumor-irradiated PD-L1^−/−^ mice was increased after tumor IR (Figure 4I). These results demonstrated that IR combined with PD-L1 deficiency promoted the proliferation of CD8^+^ T cells and its differentiation into memory T cells.

### PD-L1 blockage or knockdown enhanced the response of IR-induced cGAS-STING signaling and T cell activation

**Figure 5:**
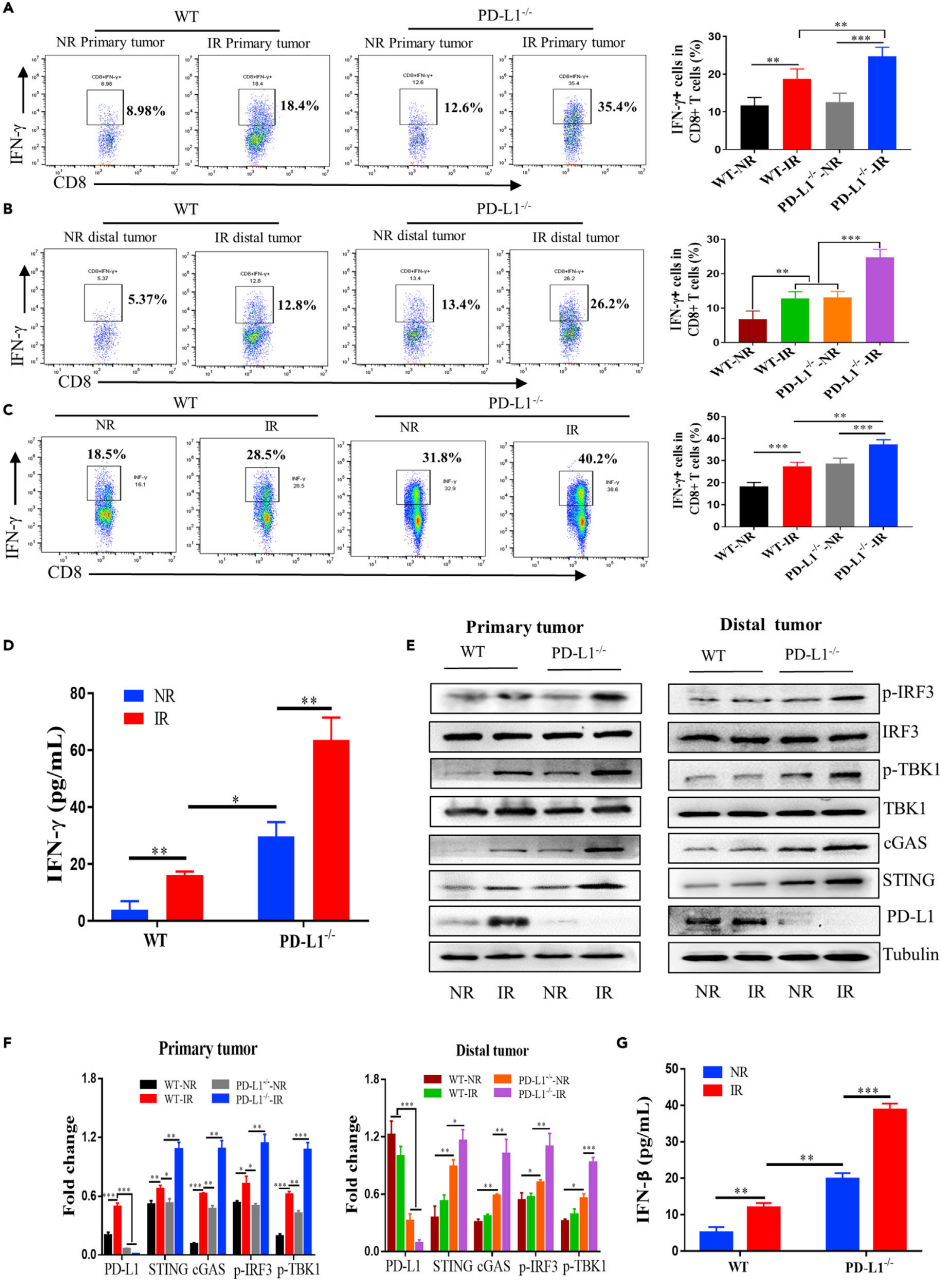
Blocking of PD-L1 enhanced the response of IR-induced cGAS-STING signaling Primary tumor in PD-L1 wt and PD-L1^−/−^ mice was irradiated (IR) or non-irradiated (NR). (A and B) Flow cytometric analysis of the percentage of IFN-γ positive cells in CD8^+^ T cells (n = 5 mice for each group). (C) Flow cytometric analysis of the percentage of IFN-γ positive cells in CD8^+^ T cells of spleen (n = 4 mice for each group). (D) ELISA assay of the concentration of IFN-γ in mice serum (n = 3 mice for each group). (E and F) Western blot assay of the expression levels of p-IRF3, IRF3, p-TBK1, TBK, cGAS, and PD-L1 in primary and distal tumors. (G) ELISA assay of the concentration of IFN-β in mice serum. n = 3 mice for each group.

It has been known that IFN-γ could enhance the toxic function and motility of CD8^+^ T cells (Bhat et al., 2017). Figure 5 illustrates that the expression levels of IFN-γ in CD8^+^ T cells of both primary and distal tumors were significantly increased after IR, and they had the highest levels in the irradiated tumor of PD-L1^−/−^ mice (Figures 5A and 5B). Meanwhile, the IFN-γ expression in intrasplenic CD8^+^ T cells was also increased by IR and was more profound in PD-L1^−/−^ mice (Figure 5C). ELISA assay revealed that the concentration of serum IFN-γ was extensively increased after tumor irradiation (Figure 5D). Notably, the concentration of IFN-γ in the serum of PD-L1^−/−^ mice was much higher than that in PD-L1 wt mice even with tumor IR. Accordingly, the release of IFN-γ from CD8^+^ T cells could be enhanced by PD-L1 deficiency and contributed to radiation-induced ATAE.

On other hand, radiation-induced DNA fragments can activate cGAS-STING pathway associated with inflammation and innate immunity (Li et al., 2020; Wang et al., 2020). In consistent, we found that the protein expressions of STING, cGAS, p-IRF3, p-TBK1, and PD-L1 were increased in the irradiated tumor although the protein expressions of IRF3 and TBK1 were not increased. Except PD-L1, they had much high levels in PD-L1^−/−^ mice in comparison with PD-L1 wt mice. In the distal tumor, the expressions of these proteins of cGAS-STING pathway had no changes after IR in PD-L1 wt mice but were still obviously increased in PD-L1^−/−^ mice after IR (Figures 5E and 5F). Because IRF3 could regulate the expression of type-I interferon, we detected the IFN-β level in the serum of mice and found that it was increased after tumor IR and had much a high level in PD-L1^−/−^ mice (Figure 5G), which was similar to the radiation response of IFN-γ production in mice serum (Figure 5D).

**Figure 6:**
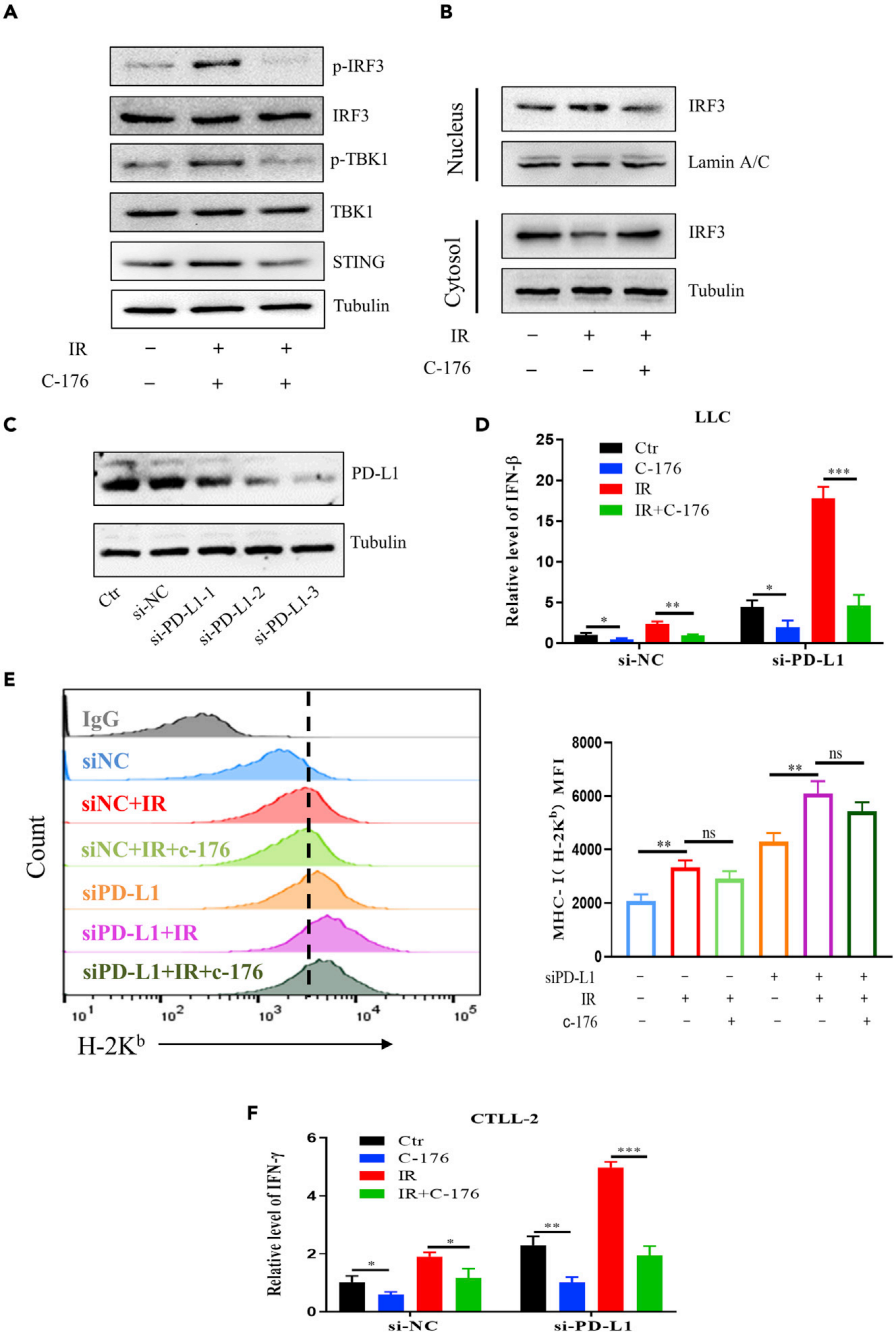
Inhibition of cGAS-STING signaling in LLC cells attenuated the activation of T cells (A) Western blot assay of p-IRF3, IRF3, p-TBK1, TBK1, and STING in the untreated and irradiated LLC cells treated with or without C-176 (a STING inhibitor). (B) Western blot assay of cytosolic and nuclear IRF-3 in the indicated group as Figure 6A. (C) Western blot assay of PD-L1 in LLC cells treated with different si-PD-L1 for 36 h. The most effective si-PD-L1-3 was used as si-PD-L1 for further PD-L1 silence treatment. (D) RT-qPCR measurement of IFN-β mRNA in LLC cells treated with C-176. PD-L1 was knocked down 36 h prior to drug treatment using si-PD-L1. Cells were irradiated with 4 Gy X-rays (IR) or not (Ctr). (E) Flow cytometry assay of the expression of MHC-I (H-2K^b^) on the surface of LLC cells under indicated treatment as Figure 6D, and the MFI value of each group was represented on the right plot. (F) RT-qPCR measurement of IFN-γ mRNA in CTLL-2 cells that were co-cultured with LLC cells under indicated treatment as Figure 6D. ∗p < 0.05, ∗∗p < 0.01, ∗∗∗p < 0.001 between indicated groups.

Next, we co-cultured LLC cells with murine T cells CTLL-2 to confirm whether the release of IFN-γ from T lymphocytes was dependent on the expression of IFN-β in LLC cells through cGAS-STING signal pathway. With the treatment of STING inhibitor C-176, the protein expression levels of STING, p-TBK1, and p-IRF3 were effectively suppressed in the irradiated LLC cells (Figure 6A). Moreover, it was found that, after irradiation, the expression of nuclear IRF3 protein was increased and the expression of cytoplasmic IRF3 was reduced, indicating the nuclear transport of IRF3. But this protein transport was inhibited by C-176 (Figure 6B). Then, we used the mouse PD-L1-targeting siRNA (si-PD-L1) to knockdown the expression of PD-L1 in LLC cells (Figure 6C) and detected IFN-β expression in LLC cells. After IR, the mRNA level of IFN-β was gently increased but extensively increased in LLC cells with si-PD-L1 transfection (Figure 6D). However, under the treatment of C-176, the mRNA levels of IFN-β were all abrogated in the irradiated LLC cells with different PD-L1 status. Further studies revealed that IR increased the expression of MHC-I (H-2K^b^) on the surface of LLC cells, which was more profound in the si-PD-L1-transfected cells, and these increases were attenuated by C-176 (Figure 6E). Moreover, cell co-culture experiments showed that murine T cells CTLL-2 could be activated by the irradiated bystander LLC cells and thus had an elevated level of IFN-γ mRNA. This radiation-induced bystander response (RIBE) became more profound when LLC cells were transfected with si-PD-L1 but it was diminished by STING inhibitor C-176 (Figure 6F), indicating that the release of IFN-β exerted an important influence on the activation of T cells. Taken together, inhibition of cGAS-STING pathway could impair the expression of IFN-β in the irradiated tumor cells, which resulted in the decrease of IFN-γ production in the bystander T lymphocytes.

### Inhibition of autophagy amplified antitumor effect induced by IR combined with PD-L1 deficiency

**Figure 7:**
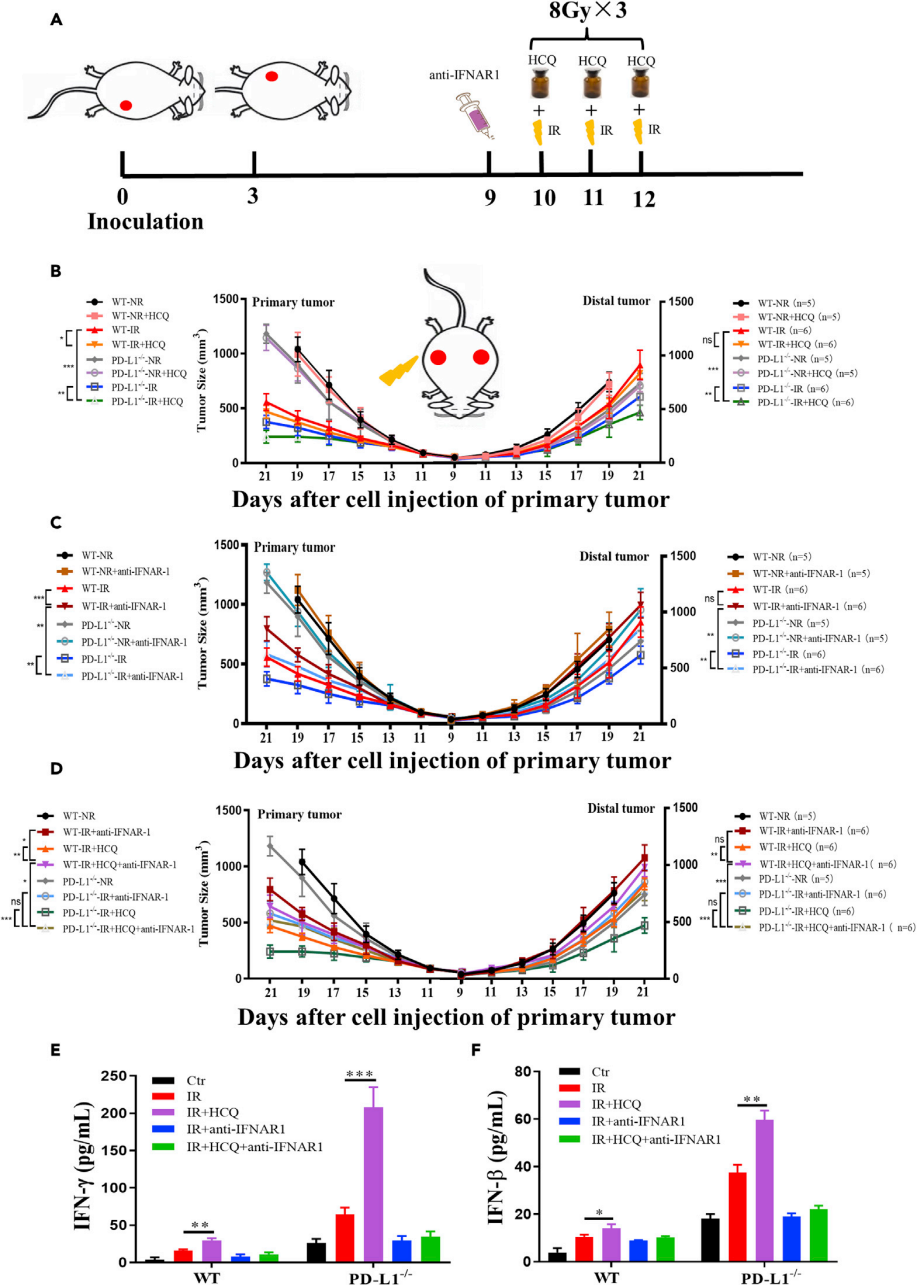
Inhibition of autophagy enhanced IR-induced antitumor abscopal effect (A) Schema of the mice model for the investigation of the role of autophagy in ATAE. Primary tumor in PD-L1 wt and PD-L1^−/−^ mice was irradiated (IR) or non-irradiated (NR). HCQ (autophagy inhibitor) (50 mg/kg) was injected intraperitoneally 2 h before each IR, anti-IFNAR1 were injected intraperitoneally one day before first IR. (B) The tumor growth curves of primary and distal tumors in indicated groups including HCQ treatment. (C) The tumor growth curves of primary and distal tumors in the indicated groups including anti-IFNAR1 treatment. (D) The tumor growth curves of primary and distal tumors in indicated groups including the combination treatment of HCQ and anti-IFNAR1. (E and F) ELISA assay of the concentration of IFN-γ (E) and IFN-β (F) in mice serum of indicated group. ns, not statistically significant, ∗p < 0.05, ∗∗p < 0.01, ∗∗∗p < 0.001 between indicated groups. n = 10 mice for each group.

Autophagy has been reported to contribute to the immune evasion of pancreatic cancer and glioma (Ruan et al., 2019; Yamamoto et al., 2020). To know whether autophagy was involved in the radiation-induced ATAE, we administrated hydroxychloroquine (HCQ) solution into mice during fractional IR to dampen autophagy incidence (Figure 7A). It was found that that this HCQ administration further enhanced tumor growth inhibition induced by IR, which was more effective in PD-L1^−/−^ mice. Moreover, IR combined with HCQ also effectively inhibited the growth of non-irradiated distal tumors in PD-L1^−/−^ mice (Figure 7B).

Moreover, we administrated anti-IFNAR1 into mice one day before IR to block the type-I interferon signaling factor (Figure 7A). Figure 7C illustrates that this administration impaired the retard of tumor growth caused by IR both in primary and distal tumors, and the superiority of the growth inhibition of distal tumor in PD-L1^−/−^ mice was also reversed. Furthermore, in the presence of anti-IFNAR1, the inhibition effect of IR combined with HCQ on tumor growth was impaired, whereas the reversal effect of tumor growth inhibition was more evident in primary and distal tumors in PD-L1^−/−^ mice due to the blockade of IFN-β signals (Figure 7D). In addition, HCQ enhanced the secretion of IFN-β and IFN-γ in the serum of mice with IR on primary tumor, and this elevation was more significant in PD-L1^−/−^ mice. But these enhancement of INFs secretion was eliminated by anti-IFNAR1 especially in PD-L1^−/−^ mice (Figures 7E and 7F).

In addition, flow cytometric analysis of tumor tissue showed that IR on primary tumor promoted more obvious aggregations of intratumoral CD8^+^ T cells in both primary and distal tumors in PD-L1^−/−^ mice pretreated by HCQ, and correspondingly, much IFN-γ was released from CD8^+^ T cells. But this combination of IR and HCQ induced increases of CD8^+^ T cells and IFN-γ in mice were diminished by anti-IFNAR1 (Figure S2A and S2B). We also photographed and weighed the spleens of PD-L1 wt and PD-L1^−/−^ mice after primary tumor IR and found that tumor-caused splenomegaly was significantly relieved by IR and HCQ treatment, but this retardation was partly reversed by administration of anti-IFNAR1 (Figures S3A and S3B).

Collectively, these results demonstrated that HCQ enhanced the immune response induced by IR combined with PD-L1 deficiency by amplifying IFN-β signaling, thereby intensifying the growth of primary and distal tumors.

### Chloroquine and PD-L1 deficiency enhanced the activation of cGAS-STING pathway and the cytotoxic function of T cells

**Figure 8:**
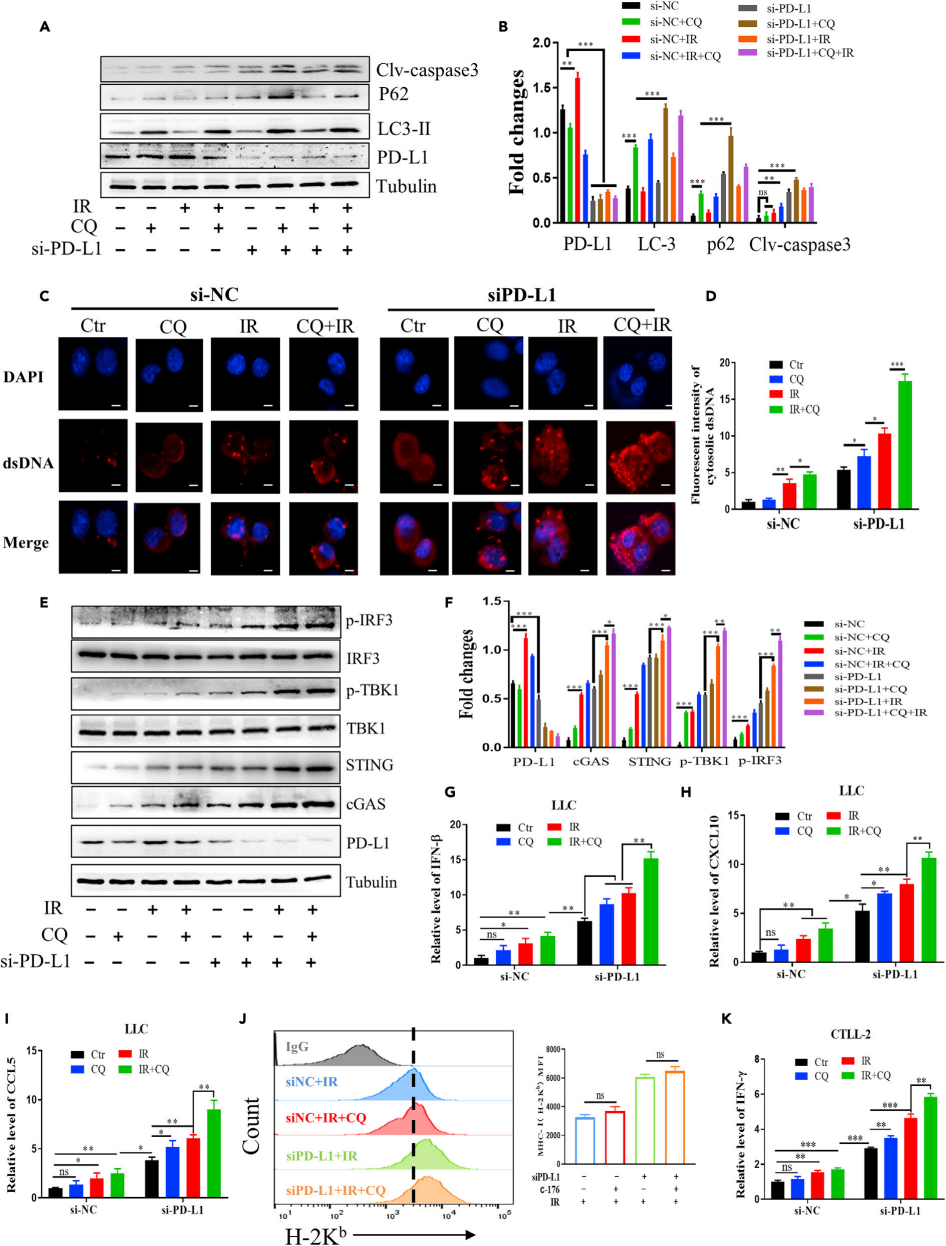
IR combined with PD-L1 silencing and CQ treatment activated cGAS-STING pathway and the function of cytotoxic T lymphocytes (A and B) Western blot assay of cleaved-caspase3, Beclin-1, P62, LC3, and PD-L1 in LLC cells treated with IR, CQ, and si-PD-L1 (A) and their expression levels related to Tubulin (B). (C and D) Representative images (C) and fluorescence density (D) of cytosolic dsDNA in LLC cells treated with IR, CQ, and si-PD-L1. Scale bars, 5 μm. (E and F) Western blot assay of the expression level of p-IRF3, p-TBK1, STING, cGAS, and PD-L1 in LLC cells treated with IR, CQ, and si-PD-L1 (E) and their expression levels related to Tubulin (F). (G–I) RT-qPCR analysis of IFN-β, CXCL10, and CCL5 mRNA in LLC cells after the treatment of IR, CQ, and si-PD-L1. (J) Flow cytometry assay of the expression of MHC-I (H-2K^b^) on the surface of LLC cells under the treatment of IR, CQ, and si-PD-L1, and the MFI value was represented on the right plot. (K) RT-qPCR analysis of IFN-γ mRNA in CTLL-2 cells that co-cultured with LLC cells after the treatment of IR, CQ, and si-PD-L1. ns, not statistically significant, ∗p < 0.05, ∗∗p < 0.01, ∗∗∗p < 0.001 between indicated groups.

Chloroquine is a widely used inhibitor of autophagy. Before usage, we checked its influence on the marker protein LC3 of autophagy by WB assay, using bafilomycin as a positive control. Figure S4 shows that treatment of LCC cells with 25 μM for 1 h could significantly increase the expressions of LC3, in consistent with other literatures (Liang et al., 2019; Chen et al., 2020). Figure 8 illustrates that, after IR, the expressions of P62, LC3-II, and Caspase-3 were increased, indicating an inhibition of autophagy and induction of apoptosis. CQ treatment alone inhibited autophagy by accumulating P62 protein and retained LC3-II in autophagosomes, and this CQ treatment also enhanced above radiation-induced protein alterations. When PD-L1 gene was knocked down by si-PD-L1 transfection, the expressions of P62, LC3-II, and Caspase-3 in CQ-treated cells were further elevated, demonstrating that the inhibition of autophagy and induction of apoptosis by CQ could be reinforced by deletion of PD-L1.

DNA damage is the most important radiation event contributing to autophagy and apoptosis. It was observed that dsDNA fragments could be aggregated in cytoplasm of LLC cells irradiated with 4 Gy of X-rays, and this dsDNA accumulation was enhanced by CQ treatment. Moreover, when PD-L1 gene was knocked down, the cytoplasmic dsDNA fragments induced by CQ, IR, and the combination of CQ + IR were further increased (Figures 8C and 8D). Because cytoplasmic dsDNA could activate the cGAS-STING pathway (Kwon and Bakhoum, 2020), we investigated the influence of CQ and PD-L1 in radiation-induced expressions of cGAS-STING related proteins in LLC cells. It was found that the protein expressions of cGAS, STING, p-IRF3, and p-TBK1 in the irradiated cells were significantly increased by CQ, and all of them had much higher levels in the PD-L1 knockdown cells (Figures 8E and 8F).

To know the influence of CQ and PD-L1 on immunoactive cytokines, we detected mRNA expressions of IFN-β and IRF-regulated chemokine (CXCL10 and CCL5) (Herzig et al., 2012; Kunz et al., 1999) in LLC cells. It was found that these cellular mRNA levels were orderly increased by CQ, IR, and the combination of CQ and IR. They were all elevated in the PD-L1 knockdown cells. Importantly, knockdown PD-L1 further promoted the expressions of IFN-β, CXCL10, and CCL5 in LLC cells treated by CQ and/or IR (Figures 8G–8I). Moreover, it was found that the treatment of CQ had no significant influence on the expression of MHC-I (H-2K^b^) induced by IR, and there was no significant difference in MHC-I (H-2K^b^) expression after the knockdown of PD-L1 (Figure 8J).

We then validated whether T cells could be activated by the upregulation of IFN-β, CXCL10, and CCL5. Thus, we co-cultured LLC cells with mouse cytotoxic T cells CTLL-2 and detected the mRNA level of IFN-γ in CTLL-2 cells. It was found that the expression of IFN-γ in CTLL-2 cells was significantly increased by the bystander irradiated LLC cells, indicating the activation of T cells. When LLC cells were treated with CQ before IR, the activation of bystander CTLL-2 cells was further enhanced. Moreover, the above CQ-mediated RIBE of IFN-γ expression in CTLL-2 cells became much more remarkable when PD-L1 gene in LLC cells was knocked down by siRNA (Figure 8K).

## Discussion

The latest advances aimed at improving RT efficiency have acknowledged the central role of immune system in the antitumor process (Liang et al., 2017). RT combined with immunotherapy has been extensively studied in cancer treatment; however, the mechanisms of radioimmunotherapy in ATAE are still obscure. Here, we reported that RT coupled with PD-L1 deficiency inhibited the tumor growth and promoted the occurrence of ATAE by activating CD8^+^ T cells-mediated immune regulation. We found for the first time that administration of autophagy inhibitor enhanced cell killing in both irradiated primary tumor and non-irradiated abscopal tumor, which was much more profound in PD-L1^−/−^ mice due to the increased secretion of IFN-γ from intratumoral CD8^+^ T cells. Mechanistically, IR induced cytoplasmic dsDNA and activated cGAS-STING pathway in tumor cells that could release immunoactive cytokines and chemokines including IFN-β, CXCL10, and CCL5 to trigger bystander/abscopal T cells to release IFN-γ and inhibit tumor growth, especially under the situation of PD-L1 deficiency.

PD-L1 has been generally recognized as a pertinacious molecule contributing to tumor evasion (Lv et al., 2021; Ricklefs et al., 2018). Clinical cases have confirmed the positive effect of IR combined with PD-1/PD-L1 or CTLA-4 inhibitors in controlling distal metastases (Postow et al., 2012; Tsui et al., 2018), but its specific immunological mechanisms are still limitedly understood. It is well known that irradiated tumors can release neoantigens, damage-associated molecular patterns (DAMPs), and chemokines to induce CD8^+^ T cells priming, and the activated CD8^+^ T cells can migrate and infiltrate into distal tumors when a certain amount was reached (Zhao and Shao, 2020; Shiraishi et al., 2008). However, IR could also increase the expression of PD-L1 on the surface of tumor cells (Deng et al., 2014; Sheng et al., 2020), which could inhibit T cell activity and engender immune escape of tumor cells (Jiang et al., 2019; Peng et al., 2019), consequently may impair IR-induced ATAE. Increasing evidence has demonstrated that the combination of IR and immune checkpoint blockade (ICB) can extend RT-induced antitumor effect to distal tumor (Liu et al., 2018; Thangamathesvaran et al., 2018). In consistent, our current data demonstrated that PD-L1 deficiency strengthened the growth inhibitory effect on both irradiated tumor and distal tumor. We also found that with the rapid growth of tumors, the degree of splenomegaly became more serious due to the occurrence of massive extramedullary hematopoietic foci and more mature neutrophil infiltration. However, IR combined with PD-L1 deficiency effectively controlled the abnormal enlargement of the spleen and the damage to T lymphocytes, indicating that the combination of IR and PD-L1 deficiency also had a great effect on maintaining spleen function.

With respect to immunoactive molecules, it has been known that the IFN-γ can stimulate the production of potent chemoattractants and actuate T cells to the site of inflammation (Dufour et al., 2002; Kursunel and Esendagli, 2016) or enhance the migrate ability of CD8^+^ T cells to the site of antigen presentation cells (Bhat et al., 2017). Our data also demonstrated that, in comparison with intratumoral CD8^+^ T cells recruitment in PD-L1 wt mice, IR could activate more intratumoral CD8^+^ T cells in PD-L1^−/−^ mice and thus to release much IFN-γ signaling factors into blood circulatory system, which could recruit more and more CD8^+^ T cells into tumors. This loop of T cells and signaling factors promoted much stronger ATAE in PD-L1^−/−^ mice after local tumor IR.

Moreover, we found that, after IR, the proportion of CD44^hi^CD62L^low^ effector T cells or memory-like CD8^+^ T cells was increased in both primary and distal tumors, and especially had a high amount in PD-L1^−/−^ mice. It has been known that memory-like CD8^+^ T cells can continually exist in the absence of antigen stimulation, maintain the unique phenotype and higher precursor frequency, and can deliver an intensified response when reencounter the same antigens (Samji and Khanna, 2017). Therefore, the increase in the ratio of effective memory-like CD8^+^ T cells in the abscopal tumors indicates a relatively long-lasting immune reaction, which should be involved in the above loop of T cells and signaling factors and thus contributed to IR-induced ATAE. Similarly, the intratumoral immune response induced by IR combined with PD-L1 deficiency also greatly improved the memory-like CD8^+^ T cells response in the spleen, which further promoted the long-term immune surveillance.

On the other hand, the increased level of autophagy led to the radioresistance of tumors (Digomann et al., 2019), and the autophagy inhibition combined with PD-L1 depletion could improve tumor treatment efficiency (Ruan et al., 2019; Zhang et al., 2021). We confirmed that autophagy inhibitor might be able to promote IR-induced ATAE in PD-L1^−/−^ mice. It was reported that the enhancement effect of ICB on IR-induced abscopal effect was dependent on cGAS/STING (Vanpouille-Box et al., 2017). The cell-intrinsic cGAS-STING signaling pathway enhanced the persistence and expansion of adoptively transferred T cells (Li et al., 2020). For impeding the misincorporation into the genome, DNA fragments could be exported from nuclear actively, and then received by the cytoplasmic sensors to trigger the cGAS-STING pathway and activate the immune response (Parkes et al., 2017; Ding et al., 2018). Our data showed that IR could trigger cGAS-STING pathway by inducing the accumulation of dsDNA in the cytoplasm, which was more profound in PD-L1^−/−^ mice. Therefore, PD-L1 silencing enhanced the activation of cGAS-STING signaling pathway stimulated by IR.

It was reported that cGAS-STING pathway could be restrained by mitophagy-mediated mtDNA clearance (Bai and Liu, 2019). But our results showed that, when autophagy was inhibited by CQ/HCQ, dsDNA fragments were presented more obvious aggregation in the cytoplasm of LLC cells and strengthened the protein expression levels of cGAS-STING pathway in the irradiated cells, and finally enhanced tumor IR-induced ATAE.

How was the radiation signaling transmitted to bystander/abscopal T cells? The activation of cGAS-STING in cancer cells can enhance the production of type I interferon which then exerted potent effects on the priming of antitumor T cells (Ablasser and Chen, 2019; Barber, 2015). Our data showed that when the type I interferon receptor was blocked by anti-IFNAR1, the concentration of irradiated tumor-released IFN-β in mice serum was eliminated, which might weaken the activation of intratumoral CD8^+^ T cells and therefore reduced the release of IFN-γ, leading to the inhibition of IR-induced ATAE whether PD-L1 was knocked down or autophagy was inhibited by HCQ. Therefore, the production of IFN-β by cancer cells was required for priming abscopal responses by promoting the expansion of CD8^+^ T cells (Vanpouille-Box et al., 2017; Formenti et al., 2018).

In summary, this study provides an unexploited functional and mechanistic insights of tumor growth control and abscopal effect promotion by IR combined with autophagy inhibition in PD-L1 deficiency mouse host. We propose a closed-loop process of which tumor IR combined with host PD-L1 depletion increases the recruitment of CD8^+^ T cells that could differentiate into effective memory T cells and secret IFN-γ to challenge the tumors. After autophagy inhibitor being used in union, much more IR-induced dsDNA fragments are restrained and accumulated in the cytoplasm of cells, especially with PD-L1 silencing, which elevates radiation-activated cGAS-STING pathway and further enhances the above process of immune antitumor. In view of the outstanding outcome of oncotherapy in the mice model, anti-PD-L1/PD-1 monoclonal antibodies and autophagy inhibitor CQ/HCQ have been ratified for clinical treatment of patients with cancer. The combination of IR with PD-L1 depletion and autophagy inhibition has far-reaching guiding significance in the cure of primary tumors and metastases in clinic.

### Limitations of the study

Our findings demonstrate that IR combined with PD-L1 deficiency strikingly activates CD8^+^ T cells that contribute to ATAE through cGAS-STING signaling pathway. However, we did not sort out the CD8^+^ T cells infiltrated in tumor to explore the changes of signaling pathway in CD8^+^ T cells. In addition, we did not further investigate the recruitment route of activated T cells toward distant tumors. Therefore, further studies on the signal transduction of CD8^+^ T cells as well as the motility of activated CD8^+^ T cells may help us to better understand the occurrence of ATAE in tumor microenvironment.

## STAR★Methods

### Key resources table

**Table:** 

REAGENT or RESOURCE	SOURCE	IDENTIFIER
**Antibodies**		

Rabbit monoclonal Anti-PD-L1	Abcam	Cat # ab213480 RRID: AB_2773715
Rabbit monoclonal Anti- cGAS	Abcam	Cat # ab252416 N/A
Rabbit monoclonal Anti- p-IRF3	Cell Signaling Technology	Cat # 4947 RRID: AB_823547
Rabbit monoclonal Anti- p-TBK1	Cell Signaling Technology	Cat # 5483 RRID: AB_10693472
Rabbit monoclonal Anti- IRF-3	Cell Signaling Technology	Cat # 11904 RRID: AB_2722521
Rabbit monoclonal Anti- TBK1	Cell Signaling Technology	Cat # 38066 RRID: AB_2827657
Rabbit monoclonal Anti- LC3	Cell Signaling Technology	Cat # 4599 RRID: AB_10548192
Rabbit monoclonal Anti-p62	Cell Signaling Technology	Cat # 5114 RRID: AB_10624872
Rabbit monoclonal Anti-Cleaved Caspase-3	Cell Signaling Technology	Cat # 9664 RRID: AB_2070042
Rabbit Polyclonal Anti-STING	Proteintech	Cat #19851-1-AP RRID: AB_10665370
Rabbit Polyclonal Anti- Lamin A/C	Proteintech	Cat # 10298-1-AP RRID: AB_2296961
Rabbit Polyclonal Anti-α-Tubulin	Beyotime	Cat # AF0001
FITC anti-mouse CD3ε	Biolegend	Cat # 100306 RRID: AB_312671
PE anti-mouse CD4	Biolegend	Cat # 100408 RRID: AB_312693
APC anti-mouse CD8a	Biolegend	Cat # 100712 RRID: AB_312751
PE/Cyanine7 anti-mouse NK-1.1	Biolegend	Cat # 108713 RRID: AB_389363
PE anti-mouse CD279 (PD-1)	Biolegend	Cat # 135205 RRID: AB_1877232
PE anti-mouse Ki-67	Biolegend	Cat # 652403 RRID: AB_2561524
PE/Cyanine7 anti-mouse/human CD44	Biolegend	Cat # 103029 RRID: AB_830786
APC/Cyanine7 anti-mouse CD62L	Biolegend	Cat # 104427 RRID: AB_830798
PE/Cyanine7 anti-mouse IFN-γ	Biolegend	Cat # 505825 RRID: AB_1595591
APC anti-mouse H-2K^b^/H-2D^b^	Biolegend	Cat # 114613 RRID: AB_2750193
Purified anti-mouse IFNAR-1	Biolegend	Cat # 127322 RRID: AB_11149116
dsDNA (rDSD/4565)	NOVUS	Cat # NBP3-07670

**Chemicals, Peptides, and Recombinant Proteins**		

Chloroquine	MedChemExpress	Cat # HY-17589A
Hydroxychloroquine sulfate	MedChemExpress	Cat # HY-B1370
Bafilomycin A1	MedChemExpress	Cat # HY-100558
Collagenase IV	YEASEN	Cat # 40510ES60
Deoxyribonuclease I (DNase I)	YEASEN	Cat # 10607ES15
Trizol	Invitrogen	Cat # 15596026
Red Blood Cell Lysis Buffer	Beyotime	Cat # C3702
Mouse Direct PCR Kit	Bimake	Cat # B40013
Fixation Buffer	Biolegend	Cat # 420801
Intracellular Staining Permeabilization Wash Buffer	Biolegend	Cat # 421002
Cell Stimulation Cocktail	Invitrogen	Cat # 00-4975-93
2x M-PCR OPTI™ Mix (Dye Plus)	Bimake	Cat # B45012

**Critical Commercial Assays**		

riboFECT CP Transfection Kit	RiboBio	Cat # C10511-05
True-Nuclear™ Transcription Factor Buffer Set	Biolegend	Cat # 424401
FastKing1st strand cDNA Synthesis Kit	TIANGEN	Cat # KR118
SuperReal PreMix Plus (SYBR Green)	TIANGEN	Cat # FP205
Nuclear and Cytoplasmic Protein Extraction Kit	Beyotime	Cat # P0028

**Experimental models: cell lines**		

LLC cell lines	American Type Culture Collection	Cat # CRL-1642 RRID:CVCL_4358
CTLL-2 cell lines	American Type Culture Collection	Cat # TIB-214 RRID:CVCL_0227

**Oligonucleotides**		

IFN-β forward: CGTGGGAGATGT CCTCAACT	This paper	N/A
IFN-β reverse: CCTGAAGATCTC TGCTCGGAC	This paper	N/A
CXCL10 forward: AGTGCTGCCGTC ATT TTC TG	This paper	N/A
CXCL10 reverse: ATCTCAACACGT GGGCAGG	This paper	N/A
CCL5 forward: CACCATATGGCT CGGACACC	This paper	N/A
CCL5 reverse: TCTGGGTTGGCA CACACTTG	This paper	N/A
β-actin forward: AGAAGCTGTGCT ATGTTGCTCTA	This paper	N/A
β-actin reverse: AGACAGCACTGT GTTGGCATA	This paper	N/A
IFN-γ forward: CCACGGCACAGT CATTGAAA	This paper	N/A
IFN-γ reverse: TTGCTGATGGCC TGATTGTCT	This paper	N/A

**Software and Algorithms**		

ImageJ	ImageJ	N/A
GraphPad Prism 8	GraphPad	N/A
FlowJo	Treestar	RRID: SCR_008520

**Other**		

Dulbecco’s Modified Eagle Medium	Gibco	Cat # 11965118
Fetal bovine serum	Gibco	Cat # 16000-044

### Resource availability

#### Lead contact

Further information and requests for resources and reagents should be directed to and will be fulfilled by the lead contact, Chunlin Shao (clshao@shum.edu.cn)

#### Material availability

Antibodies were obtained from the commercial or academic sources described in the STAR Methods key resources table. Material generated in this study will be made available upon reasonable request.

### Experimental models and subject details

#### Cell culture

Lewis lung carcinoma (LLC) cell line and murine T cell line of CTLL-2 were purchased from the American Type Culture Collection (ATCC, Manassas, VA). They were cultured in DMEM supplemented with 10% FBS and 1% penicillin/streptomycin and extra 20 IU/mL of IL-2 for CTLL-2 cells and maintained in a humidified atmosphere containing 5% CO_2_ at 37°C. All cell culture reagents were purchased from GIBCO (Invitrogen, Grand Island, NY).

#### Animals

C57BL/6J mice with PD-L1 wt and PD-L1^−/−^ were purchased from Shanghai Model Organisms Center. The PD-L1 status of mice were confirmed before each experiment. All experimental male animals were conducted 7-8 weeks of age, and its protocol was approved by the Animal Welfare and Ethics Committee of Fudan University (No. 20171304A215).

### Method details

#### LLC syngeneic tumor construction and treatment

LLC cells (2×10^6^ in 100 μL PBS) were subcutaneously injected into the right flank (as primary tumor) on day 0 and in the left flank (as abscopal tumor) on day 3 of each male mouse with different PD-L1 status. The tumor volume on both flanks was measured every two days after injection and calculated with a formula V = (width^2^×length)/2. When tumor volume reached to about 50 mm^3^, the primary tumor was locally exposed to X-rays (X-RAD 320, PXI, USA) with 24 Gy in 3 fractions in 3 successive days (day 10, 11, and 12 after cell injection).

For the treatment with an autophagy inhibitor hydroxychloroquine (HCQ) (MedChemExpress, Monmouth Junction, NJ, USA), HCQ (50 mg kg^−1^ in 100 μL saline) or its saline control was injected intraperitoneally (i.p.) into tumor-bearing mice at 2 h before each IR. For blocking type I interferon (IFN-I), anti-IFNAR1 (500 μg, BioLegend, San Diego, CA) was administrated intraperitoneally into each mouse on day 9 of cell injection i.e., one day before tumor IR.

#### Tissue collection and flow cytometry assay

After 21 days from the first tumor inoculation, mice syngeneic tumor and spleens were collected. Preparation of mouse tumor cells: Tumors were diced and digested with a medium containing 1-2 mg/mL collagenase IV and 10 μg/mL DNase (Yeasen Biotechnology Co. Ltd., Shanghai, China) in a water bath at 37°C for 1 h, then the tissue homogenates were filtered with a 70 μm filter membrane. The supernatants were centrifuged at 400 g for 5 min to collect cell pellets, treated with red blood cell lysate (Beyotime Biotech., Haimen, China) for 1-2 min, then centrifuged and washed with PBS buffer containing 2% FBS. Preparation of mouse splenocytes: The spleen was gently grinded in the medium and filtered with 70 μm filter membrane, Centrifuge at 400 g for 5 min to collect the cell pellet and remove the red blood cell with red blood cell lysate for 1-2 min, then centrifuge and wash with PBS buffer containing 2% FBS.

Anti-CD3 (2 μg/mL), anti-CD4 (1 μg/mL), anti-CD8 (1 μg/mL), anti-NK1.1 (1 μg/mL), anti-CD44 (1 μg/mL), anti-CD62L (1 μg/mL), anti-PD-1 (1 μg/mL), (BioLegend, San Diego, CA) were used for surface staining at 4°C in dark condition. Ki67 staining (2 μg/mL) was performed at room temperature after permeabilization and fixation with the True-Nuclear™ Transcription Factor Buffer Set (BioLegend, San Diego, CA) and subjected to flow cytometry analyses with a CytoFLEX cytometer (Beckman-Coulter, USA).

#### Flow cytometric assay of IFN-γ

Cells isolated from spleen or tumor tissues of the indicated mice were stimulated with a cell stimulation cocktail (Thermo Fisher Scientific) for 16 h, collected and labeled with CD3 and CD8 antibody, and subjected to intracellular cytokine staining after permeabilization and fixation with intracellular staining permeabilization wash buffer and fixation buffer (BioLegend, San Diego, CA). These cells were further labeled with anti-IFN-γ (2 μg/mL) to detect the release of intracellular IFN-γ in CD3^+^ CD8^+^ T cells by flow cytometry.

#### Immunohistochemistry

The tumor tissues and spleens collected from the mice were fixed in 4% paraformaldehyde, washed with PBS then transferred to 75% ethanol, embedded in paraffin for storage. Just before using, the tissue sections were dewaxed in xylenes and hydrated in absolute ethanol, 85% ethanol, 75% ethanol successively, rinsed in distilled water, and then heated in a microwave oven using citrate buffer (pH 6.0) to unmask antigens. After blocking the endogenous peroxidase activity with 3% H_2_O_2_, the slides were blocked with 3% BSA for 30 min at room temperature and incubated with primary antibodies against CD3, CD4, CD8 (Servicebio Technology Co., Ltd., Wuhan, China) overnight at 4°C in humidified box, then washed with PBS and incubated with HRP-conjugated secondary antibodies for 50 min. Subsequently, the slides were treated with the DAB substrate kit (Servicebio) and counterstained with hematoxylin, sealed with neutral balsam and photographed with a microscope.

#### RNA interference

PD-L1-targeting siRNAs for mouse cells (si-PD-L1-1, si-PD-L1-2, si-PD-L1-3) and its negative control (si-NC) were designed and synthesized by Ribobio (Guangzhou, China). LLC cells (1.0×10^5^ per well) were seeded into 6-well plates and transfected with 50 nM si-PD-L1 or its scramble control using riboFECT^TM^ CP Reagent (Ribobio, Guangzhou, China) according to the manufacturer’s instruction. After 36 h, the siRNA transfection efficiency was identified by Western blot assy.

#### Cell co-culture

LLC and CTLL-2 cells were co-cultured for 24 h to investigate the effects of irradiated LLC cells on bystander CTLL-2 cells. In brief, LLC cells with or without si-PD-L1 transfection were irradiated with 4 Gy X-rays (X-RAD 320, PXI, USA) at a dose rate of 1 Gy/min. In some experiments, LLC cells were pre-treated with 25 μM chloroquine (CQ, an inhibitor of autophagy) or 20 μM C-176 (MedChemExpress) (an inhibitor of STING) 1 h before IR. After IR, the culture medium of LLC cells was replaced and seeded in a 6-well plate, CTLL-2 cells were then added in the plate and co-cultured with LLC cells. The ratio of tumor cells and CTLL-2 cells was 1:3. After 24 h of cell co-culture, these cells were collected for further experiments.

#### ELISA assay of serum interferon

After each designed treatment, the whole blood was collected from mice orbits and the serum was separated. Then the concentrations of IFN-γ and IFN-β in mouse serum were measured using mouse IFN-γ ELISA kit (70-EK280/3-96, Multi Science, China) and mouse IFN-β ELISA Kit (70-EK2236-48, Multi Science) according to the manufacturer’s instructions.

#### Subcellular protein extraction

LLC cells were plated in 10-cm dishes and incubated at 37°C in 5% CO2. When cells reached about 70% confluence, LLC cells were irradiated with 4 Gy X-rays, control cells were not irradiated. For the cell group of IR combined with C-176, 20 μM C-176 were pre-treated 1 h before IR. Cells were harvested at 5 h post-IR, Cell cytoplasmic protein and nuclear proteins were extracted using a subcellular protein fractionation kit (Beyotime Biotech) according to the manufacturer’s instruction.

#### Western blotting assay

Total proteins were extracted from tissues and cells with precooled SDS lysis buffer containing protease inhibitor phenylmethanesulfonyl fluoride (PMSF) (Beyotime Biotech, Haimen, China). BCA Protein Assay Kit (Beyotime Biotech) was used to determine protein concentration. Lysates were separated in resolving gel and transferred to polyvinylidene difluoride (PVDF) membrane (Millipore, USA). After blocking with 5% skimmed milk for 2 h, the membrane was incubated with the primary antibodies overnight at 4°C. The superfluous primary antibody was washed away with TBST followed by the incubation of goat anti-rabbit IgG-HRP secondary antibody (Beyotime Biotech) for 2 h at room temperature. The chemiluminescence detection was finally carried out with the ChemiDocTM XRS imager (Bio-Rad, USA).

Antibodies against cGAS (ab252416), PD-L1 (ab213480) were purchased from Abcam (Cambridge, UK). Antibodies against p-IRF3 (#4947), p-TBK1 (#5483), IRF-3 (#11904S), TBK1 (#38066), LC3 (#4599), p62 (#5114), Cleaved Caspase-3 (#9664), were purchased from Cell Signaling Technology (Boston, USA). Antibodies against STING (19851-1-AP), Lamin A/C (10298-1-AP) was purchased from Proteintech (Wuhan, China). Antibodies against Tubulin (AF0001) was purchased from Beyotime Biotech.

#### Immunofluorescence assay of dsDNA

LLC cells with or without si-PD-L1 transfection were irradiated by 4 Gy X-rays under pre-treatment of CQ. After 5 h of IR, the cells were fixed by 4% paraformaldehyde for 30 min, permeabilized by 0.2% Triton X-100 for 2–5 min, blocked by 0.1% PBS-Tween solution for 30 min, and then incubated overnight with the primary antibody against dsDNA (Novus Biologicals, USA, dilution 1:200) and subsequently incubated with secondary antibodies conjugated with Alexa Fluor 555 (1:1000, Cell Signaling Technology) for 1 h. Cell nuclear was stained with 1.43 μM DAPI (Beyotime Biotech). Then cell fluorescence image was photographed with a high content screening system (Image Xpress Micro 4, Molecular Devices, San Jose, CA, USA).

#### Real time-PCR assay

At 6 h after irradiation or cell co-culture, total cellular RNA was extracted by using Trizol Reagent (Invitrogen, San Diego, CA, USA) according to the manufacturer’s instruction. The cDNA synthesis was performed using a FastKing RT Kit (with gDNase) (Tiangen Biotechnology, Beijing, China), and RT-PCR was performed with a SuperReal PreMix Plus Kit (SYBR Green) (Tiangen Biotechnology, Beijing, China) using a Stratagene MX3000P platform (Agilent Technologies, Santa Clara, CA). The sequences were as follows. IFN-β, forward: 5′-CGT GGG AGA TGT CCT CAA CT-3′, reverse: 5′-CCT GAA GAT CTC TGC TCG GAC-3′; CXCL10, forward: 5′-AGT GCT GCC GTC ATT TTC TG-3′, reverse: 5′-ATC TCA ACA CGT GGG CAG G-3′; CCL5, forward: 5′-CAC CAT ATG GCT CGG ACA CC-3′, reverse: 5′-TCT GGG TTG GCA CAC ACT TG-3′; β-actin, forward: 5′-AGA AGC TGT GCT ATG TTG CTC TA-3′, reverse: 5′-AGA CAG CAC TGT GTT GGC ATA-3′. IFN-γ, forward: 5′-CCA CGG CAC AGT CAT TGA AA-3′, reverse: 5′-TTG CTG ATG GCC TGA TTG TCT-3′.

### Quantification and statistical analysis

All analysis was performed using GraphPad Prism 8.0 software. two-way ANOVA test was used to analyze tumor growth data. Student’s *t* test was used to compare the significant difference between independent groups. Survival curves were analyzed by log-rank (Mantel–Cox) test. Data were presented as the mean ± SEM. p value less than 0.05 is considered statistically significant, and ∗p < 0.05, ∗∗p < 0.01, ∗∗∗p < 0.001.

## Data Availability

• All data reported in this paper will be shared by the lead contact upon request. • This paper does not report original code. • Any additional information required to reanalyze the data reported in this paper is available from the lead contact upon request.

## References

[bib1] Ablasser A., Chen Z.J. (2019). cGAS in action: expanding roles in immunity and inflammation. Science.

[bib2] Baginska J., Viry E., Berchem G., Poli A., Noman M.Z., van Moer K., Medves S., Zimmer J., Oudin A., Niclou S.P. (2013). Granzyme B degradation by autophagy decreases tumor cell susceptibility to natural killer-mediated lysis under hypoxia. Proc. Natl. Acad. Sci. USA.

[bib3] Bai J., Liu F. (2019). The cGAS-cGAMP-STING pathway: a molecular link between immunity and metabolism. Diabetes.

[bib4] Barber G.N. (2015). STING: infection, inflammation and cancer. Nat. Rev. Immunol..

[bib5] Bhat P., Leggatt G., Waterhouse N., Frazer I.H. (2017). Interferon-gamma derived from cytotoxic lymphocytes directly enhances their motility and cytotoxicity. Cell Death Dis..

[bib6] Brahmer J.R., Tykodi S.S., Chow L.Q., Hwu W.J., Topalian S.L., Hwu P., Drake C.G., Camacho L.H., Kauh J., Odunsi K. (2012). Safety and activity of anti-PD-L1 antibody in patients with advanced cancer. N. Engl. J. Med..

[bib7] Briceño E., Calderon A., Sotelo J. (2007). Institutional experience with chloroquine as an adjuvant to the therapy for glioblastoma multiforme. Surg. Neurol..

[bib8] Chang M.C., Chen Y.L., Lin H.W., Chiang Y.C., Chang C.F., Hsieh S.F., Chen C.A., Sun W.Z., Cheng W.F. (2018). Irradiation enhances abscopal anti-tumor effects of antigen-specific immunotherapy through regulating tumor microenvironment. Mol. Ther..

[bib9] Chen P., Wu Q., Feng J., Yan L., Sun Y., Liu S., Xiang Y., Zhang M., Pan T., Chen X. (2020). Erianin, a novel dibenzyl compound in Dendrobium extract, inhibits lung cancer cell growth and migration via calcium/calmodulin-dependent ferroptosis. Signal Transduct. Targeted Ther..

[bib10] Deng L., Liang H., Burnette B., Beckett M., Darga T., Weichselbaum R.R., Fu Y.X. (2014). Irradiation and anti-PD-L1 treatment synergistically promote antitumor immunity in mice. J. Clin. Invest..

[bib11] Digomann D., Linge A., Dubrovska A. (2019). SLC3A2/CD98hc, autophagy and tumor radioresistance: a link confirmed. Autophagy.

[bib12] Ding L., Kim H.J., Wang Q., Kearns M., Jiang T., Ohlson C.E., Li B.B., Xie S., Liu J.F., Stover E.H. (2018). PARP inhibition elicits STING-dependent antitumor immunity in brca1-deficient ovarian cancer. Cell Rep..

[bib13] Diskin B., Adam S., Cassini M.F., Sanchez G., Liria M., Aykut B., Buttar C., Li E., Sundberg B., Salas R.D. (2020). PD-L1 engagement on T cells promotes self-tolerance and suppression of neighboring macrophages and effector T cells in cancer. Nat. Immunol..

[bib14] Dufour J.H., Dziejman M., Liu M.T., Leung J.H., Lane T.E., Luster A.D. (2002). IFN-gamma-inducible protein 10 (IP-10; CXCL10)-deficient mice reveal a role for IP-10 in effector T cell generation and trafficking. J. Immunol..

[bib15] Eldredge H.B., Denittis A., Duhadaway J.B., Chernick M., Metz R., Prendergast G.C. (2013). Concurrent whole brain radiotherapy and short-course chloroquine in patients with brain metastases: a pilot trial. J. Radiat. Oncol..

[bib16] Finazzi T., Rordorf T., Ikenberg K., Huber G.F., Guckenberger M., Garcia Schueler H.I. (2018). Radiotherapy-induced anti-tumor immune response and immune-related adverse events in a case of recurrent nasopharyngeal carcinoma undergoing anti-PD-1 immunotherapy. BMC Cancer.

[bib17] Formenti S.C., Demaria S. (2009). Systemic effects of local radiotherapy. Lancet Oncol..

[bib18] Formenti S.C., Rudqvist N.P., Golden E., Cooper B., Wennerberg E., Lhuillier C., Vanpouille-Box C., Friedman K., Ferrari de Andrade L., Wucherpfennig K.W. (2018). Radiotherapy induces responses of lung cancer to CTLA-4 blockade. Nat. Med..

[bib19] Freeman G.J., Long A.J., Iwai Y., Bourque K., Chernova T., Nishimura H., Fitz L.J., Malenkovich N., Okazaki T., Byrne M.C. (2000). Engagement of the PD-1 immunoinhibitory receptor by a novel B7 family member leads to negative regulation of lymphocyte activation. J. Exp. Med..

[bib20] Gulley J.L., Arlen P.M., Bastian A., Morin S., Marte J., Beetham P., Tsang K.Y., Yokokawa J., Hodge J.W., Ménard C. (2005). Combining a recombinant cancer vaccine with standard definitive radiotherapy in patients with localized prostate cancer. Clin. Cancer Res..

[bib21] Herzig D.S., Driver B.R., Fang G., Toliver-Kinsky T.E., Shute E.N., Sherwood E.R. (2012). Regulation of lymphocyte trafficking by CXC chemokine receptor 3 during septic shock. Am. J. Respir. Crit. Care Med..

[bib22] Huang R.X., Zhou P.K. (2020). DNA damage response signaling pathways and targets for radiotherapy sensitization in cancer. Signal Transduct. Targeted Ther..

[bib23] Jiang X., Wang J., Deng X., Xiong F., Ge J., Xiang B., Wu X., Ma J., Zhou M., Li X. (2019). Role of the tumor microenvironment in PD-L1/PD-1-mediated tumor immune escape. Mol. Cancer.

[bib24] Juneja V.R., McGuire K.A., Manguso R.T., LaFleur M.W., Collins N., Haining W.N., Freeman G.J., Sharpe A.H. (2017). PD-L1 on tumor cells is sufficient for immune evasion in immunogenic tumors and inhibits CD8 T cell cytotoxicity. J. Exp. Med..

[bib25] Keir M.E., Liang S.C., Guleria I., Latchman Y.E., Qipo A., Albacker L.A., Koulmanda M., Freeman G.J., Sayegh M.H., Sharpe A.H. (2006). Tissue expression of PD-L1 mediates peripheral T cell tolerance. J. Exp. Med..

[bib26] Khazen R., Müller S., Gaudenzio N., Espinosa E., Puissegur M.P., Valitutti S. (2016). Melanoma cell lysosome secretory burst neutralizes the CTL-mediated cytotoxicity at the lytic synapse. Nat. Commun..

[bib27] Kim C., Wang X.D., Yu Y. (2020). PARP1 inhibitors trigger innate immunity via PARP1 trapping-induced DNA damage response. Elife.

[bib28] Kunz M., Toksoy A., Goebeler M., Engelhardt E., Bröcker E.B., Gillitzer R. (1999). Strong expression of the lymphoattractant C-X-C chemokine Mig is associated with heavy infiltration of T cells in human malignant melanoma. J. Pathol..

[bib29] Kursunel M.A., Esendagli G. (2016). The untold story of IFN-γ in cancer biology. Cytokine Growth Factor Rev..

[bib30] Kwon J., Bakhoum S.F. (2020). The cytosolic DNA-sensing cGAS-STING pathway in cancer. Cancer Discov..

[bib31] Latchman Y.E., Liang S.C., Wu Y., Chernova T., Sobel R.A., Klemm M., Kuchroo V.K., Freeman G.J., Sharpe A.H. (2004). PD-L1-deficient mice show that PD-L1 on T cells, antigen-presenting cells, and host tissues negatively regulates T cells. Proc. Natl. Acad. Sci. USA.

[bib32] Levy J.M.M., Towers C.G., Thorburn A. (2017). Targeting autophagy in cancer. Nat. Rev. Cancer.

[bib33] Li W., Lu L., Lu J., Wang X., Yang C., Jin J., Wu L., Hong X., Li F., Cao D. (2020). cGAS-STING-mediated DNA sensing maintains CD8(+) T cell stemness and promotes antitumor T cell therapy. Sci. Transl. Med..

[bib34] Liang H., Deng L., Hou Y., Meng X., Huang X., Rao E., Zheng W., Mauceri H., Mack M., Xu M. (2017). Host STING-dependent MDSC mobilization drives extrinsic radiation resistance. Nat. Commun..

[bib35] Liang L., Hui K., Hu C., Wen Y., Yang S., Zhu P., Wang L., Xia Y., Qiao Y., Sun W. (2019). Autophagy inhibition potentiates the anti-angiogenic property of multikinase inhibitor anlotinib through JAK2/STAT3/VEGFA signaling in non-small cell lung cancer cells. J. Exp. Clin. Cancer Res..

[bib36] Liauw S.L., Connell P.P., Weichselbaum R.R. (2013). New paradigms and future challenges in radiation oncology: an update of biological targets and technology. Sci. Transl. Med..

[bib37] Lin H., Wei S., Hurt E.M., Green M.D., Zhao L., Vatan L., Szeliga W., Herbst R., Harms P.W., Fecher L.A. (2018). Host expression of PD-L1 determines efficacy of PD-L1 pathway blockade-mediated tumor regression. J. Clin. Invest..

[bib38] Liu Y., Dong Y., Kong L., Shi F., Zhu H., Yu J. (2018). Abscopal effect of radiotherapy combined with immune checkpoint inhibitors. J. Hematol. Oncol..

[bib39] Lv H., Lv G., Chen C., Zong Q., Jiang G., Ye D., Cui X., He Y., Xiang W., Han Q. (2021). NAD(+) metabolism maintains inducible PD-L1 expression to drive tumor immune evasion. Cell Metab..

[bib40] Mukherjee D., Coates P.J., Lorimore S.A., Wright E.G. (2014). Responses to ionizing radiation mediated by inflammatory mechanisms. J. Pathol..

[bib41] Noman M.Z., Janji B., Kaminska B., Van Moer K., Pierson S., Przanowski P., Buart S., Berchem G., Romero P., Mami-Chouaib F., Chouaib S. (2011). Blocking hypoxia-induced autophagy in tumors restores cytotoxic T-cell activity and promotes regression. Cancer Res..

[bib42] Pardoll D.M. (2012). The blockade of immune checkpoints in cancer immunotherapy. Nat. Rev. Cancer.

[bib43] Park S.S., Dong H., Liu X., Harrington S.M., Krco C.J., Grams M.P., Mansfield A.S., Furutani K.M., Olivier K.R., Kwon E.D. (2015). PD-1 restrains radiotherapy-induced abscopal effect. Cancer Immunol. Res..

[bib44] Parkes E.E., Walker S.M., Taggart L.E., McCabe N., Knight L.A., Wilkinson R., McCloskey K.D., Buckley N.E., Savage K.I., Salto-Tellez M. (2017). Activation of STING-dependent innate immune signaling by S-Phase-Specific DNA damage in breast cancer. J. Nat.l Cancer Inst..

[bib45] Peng S., Wang R., Zhang X., Ma Y., Zhong L., Li K., Nishiyama A., Arai S., Yano S., Wang W. (2019). EGFR-TKI resistance promotes immune escape in lung cancer via increased PD-L1 expression. Mol. Cancer.

[bib46] Pfannenstiel L.W., McNeilly C., Xiang C., Kang K., Diaz-Montero C.M., Yu J.S., Gastman B.R. (2019). Combination PD-1 blockade and irradiation of brain metastasis induces an effective abscopal effect in melanoma. OncoImmunology.

[bib47] Pilones K.A., Vanpouille-Box C., Demaria S. (2015). Combination of radiotherapy and immune checkpoint inhibitors. Semin. Radiat. Oncol..

[bib48] Postow M.A., Callahan M.K., Barker C.A., Yamada Y., Yuan J., Kitano S., Mu Z., Rasalan T., Adamow M., Ritter E. (2012). Immunologic correlates of the abscopal effect in a patient with melanoma. N. Engl. J. Med..

[bib49] Ricklefs F.L., Alayo Q., Krenzlin H., Mahmoud A.B., Speranza M.C., Nakashima H., Hayes J.L., Lee K., Balaj L., Passaro C. (2018). Immune evasion mediated by PD-L1 on glioblastoma-derived extracellular vesicles. Sci. Adv..

[bib50] Rojas-Puentes L.L., Gonzalez-Pinedo M., Crismatt A., Ortega-Gomez A., Gamboa-Vignolle C., Nuñez-Gomez R., Dorantes-Gallareta Y., Arce-Salinas C., Arrieta O. (2013). Phase II randomized, double-blind, placebo-controlled study of whole-brain irradiation with concomitant chloroquine for brain metastases. Radiat. Oncol..

[bib51] Ruan S., Xie R., Qin L., Yu M., Xiao W., Hu C., Yu W., Qian Z., Ouyang L., He Q., Gao H. (2019). Aggregable nanoparticles-enabled chemotherapy and autophagy inhibition combined with anti-PD-L1 antibody for improved glioma treatment. Nano Lett..

[bib52] Samji T., Khanna K.M. (2017). Understanding memory CD8(+) T cells. Immunol. Lett..

[bib53] Sen T., Rodriguez B.L., Chen L., Corte C.M.D., Morikawa N., Fujimoto J., Cristea S., Nguyen T., Diao L., Li L. (2019). Targeting DNA damage response promotes antitumor immunity through STING-mediated T-cell activation in small cell lung cancer. Cancer Discov..

[bib54] Sheng H., Huang Y., Xiao Y., Zhu Z., Shen M., Zhou P., Guo Z., Wang J., Wang H., Dai W. (2020). ATR inhibitor AZD6738 enhances the antitumor activity of radiotherapy and immune checkpoint inhibitors by potentiating the tumor immune microenvironment in hepatocellular carcinoma. J. Immunother. Cancer.

[bib55] Shi L., Chen S., Yang L., Li Y. (2013). The role of PD-1 and PD-L1 in T-cell immune suppression in patients with hematological malignancies. J. Hematol. Oncol..

[bib56] Shiraishi K., Ishiwata Y., Nakagawa K., Yokochi S., Taruki C., Akuta T., Ohtomo K., Matsushima K., Tamatani T., Kanegasaki S. (2008). Enhancement of antitumor radiation efficacy and consistent induction of the abscopal effect in mice by ECI301, an active variant of macrophage inflammatory protein-1α. Clin. Cancer Res..

[bib57] Sotelo J., Briceño E., López-González M.A. (2006). Adding chloroquine to conventional treatment for glioblastoma multiforme: a randomized, double-blind, placebo-controlled trial. Ann. Intern. Med..

[bib58] Sun C., Mezzadra R., Schumacher T.N. (2018). Regulation and function of the PD-L1 checkpoint. Immunity.

[bib59] Tang H., Liang Y., Anders R.A., Taube J.M., Qiu X., Mulgaonkar A., Liu X., Harrington S.M., Guo J., Xin Y. (2018). PD-L1 on host cells is essential for PD-L1 blockade-mediated tumor regression. J. Clin. Invest..

[bib60] Thangamathesvaran L., Shah R., Verma R., Mahmoud O. (2018). Immune checkpoint inhibitors and radiotherapy-concept and review of current literature. Ann. Transl. Med..

[bib61] Topalian S.L., Hodi F.S., Brahmer J.R., Gettinger S.N., Smith D.C., McDermott D.F., Powderly J.D., Carvajal R.D., Sosman J.A., Atkins M.B. (2012). Safety, activity, and immune correlates of anti-PD-1 antibody in cancer. N. Engl. J. Med..

[bib62] Tsui J.M., Mihalcioiu C., Cury F.L. (2018). Abscopal effect in a stage IV melanoma patient who progressed on pembrolizumab. Cureus.

[bib63] Vanpouille-Box C., Alard A., Aryankalayil M.J., Sarfraz Y., Diamond J.M., Schneider R.J., Inghirami G., Coleman C.N., Formenti S.C., Demaria S. (2017). DNA exonuclease Trex1 regulates radiotherapy-induced tumour immunogenicity. Nat. Commun..

[bib64] Vanpouille-Box C., Formenti S.C., Demaria S. (2018). Toward precision radiotherapy for use with immune checkpoint blockers. Clin. Cancer Res..

[bib65] Wang Y., Luo J., Alu A., Han X., Wei Y., Wei X. (2020). cGAS-STING pathway in cancer biotherapy. Mol. Cancer.

[bib66] Yamamoto K., Venida A., Yano J., Biancur D.E., Kakiuchi M., Gupta S., Sohn A.S.W., Mukhopadhyay S., Lin E.Y., Parker S.J. (2020). Autophagy promotes immune evasion of pancreatic cancer by degrading MHC-I. Nature.

[bib67] Zhang D., Reyes R.M., Osta E., Kari S., Gupta H.B., Padron A.S., Kornepati A.V.R., Kancharla A., Sun X., Deng Y. (2021). Bladder cancer cell-intrinsic PD-L1 signals promote mTOR and autophagy activation that can be inhibited to improve cytotoxic chemotherapy. Cancer Med..

[bib68] Zhang F., Wei H., Wang X., Bai Y., Wang P., Wu J., Jiang X., Wang Y., Cai H., Xu T., Zhou A. (2017). Structural basis of a novel PD-L1 nanobody for immune checkpoint blockade. Cell Discov..

[bib69] Zhao X., Shao C. (2020). Radiotherapy-mediated immunomodulation and anti-tumor abscopal effect combining immune checkpoint blockade. Cancers.

