# Supplementary material for: Irradiation combined with PD-L1−/− and autophagy inhibition enhances the antitumor effect of lung cancer via cGAS-STING-mediated T cell activation
Source: iScience. 2022 Jun 30;25(8):104690. doi: 10.1016/j.isci.2022.104690 (PMC9283938; doi:10.1016/j.isci.2022.104690)
Supplement: Document S1. Figures S1–S4 [file mmc1.pdf]

iScience, Volume *25*

Supplemental information

Irradiation combined with PD-L1$^{-/-}$ and autophagy inhibition enhances the antitumor effect of lung cancer via cGAS-STING-mediated T cell activation

Xinrui Zhao, Songling Hu, Liang Zeng, Xinglong Liu, Yimeng Song, Yuhong Zhang, Qianping Chen, Yang Bai, Jianghong Zhang, Haowen Zhang, Yan Pan, and Chunlin Shao

Supplemental information

Irradiation combined with PD-L1$^{-/-}$ and autophagy inhibition enhances the antitumor effect of lung cancer via cGAS-STING mediated T cell activation

Xinrui Zhao[1#], Songling Hu[1#], Liang Zeng[1], Xinglong Liu[1], Yimeng Song[1], Yuhong Zhang[1], Qianping Chen[1], Yang Bai[1], Jianghong Zhang[1], Haowen Zhang[2]*, Yan Pan[1]*, Chunlin Shao[1]*

Running title: PD-L1 deficiency promotes radiation induced abscopal effect

[#] These authors contributed equally to this work.

Affiliations:

1. Institute of Radiation Medicine, Shanghai Medical College, Fudan University, Shanghai 200032, China.

2. State Key Laboratory of Radiation Medicine and Protection, School of Radiation Medicine and Protection, Medical College of Soochow University, Suzhou 215123, China

[*]Authors for correspondence

Prof. Chunlin Shao, Phone: +86-21-64048677, E-mail: clshao@shum.edu.cn.

Dr. Yan Pan, Phone: +86-21-64436720, E-mail: swallowpan@fudan.edu.cn

Dr. Haowen Zhang, Phone: +86-512-65880065, E-mail: hwzhang@suda.edu.cn

Lead Contact:

Prof. Chunlin Shao, Phone: +86-21-64048677, E-mail: clshao@shum.edu.cn.

Supplemental figures and legends

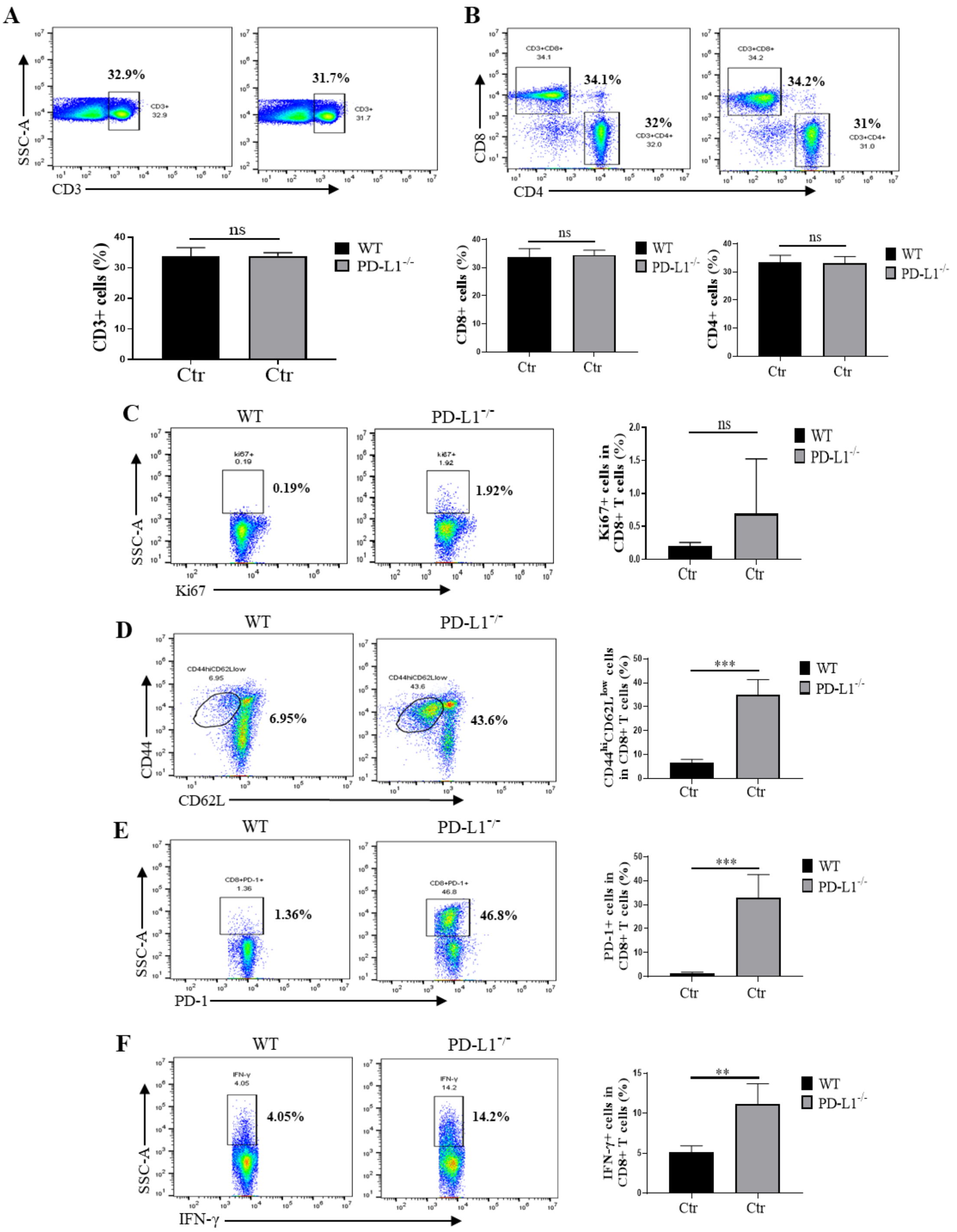

Figure S1. The basal level of immune cells and immune functions in the spleen of PD-L1 wt and PD-L1$^{-/-}$ mice, related to Figure 4.

(A, B) The percentages of CD3+ T cells, CD4+ T cells and CD8+ T cells in splenocytes were detected by flow cytometry. **(C-F)** The cell proliferation marker (Ki67), the percentage of memory-like (CD44^{hi}CD62L^{low}) T cells, the expression of PD-1 and the ability of IFN-γ induction in CD8+ T cells were assayed by flow cytometer. ** $p < 0.01$, *** $p < 0.001$. n = 4 mice each group.

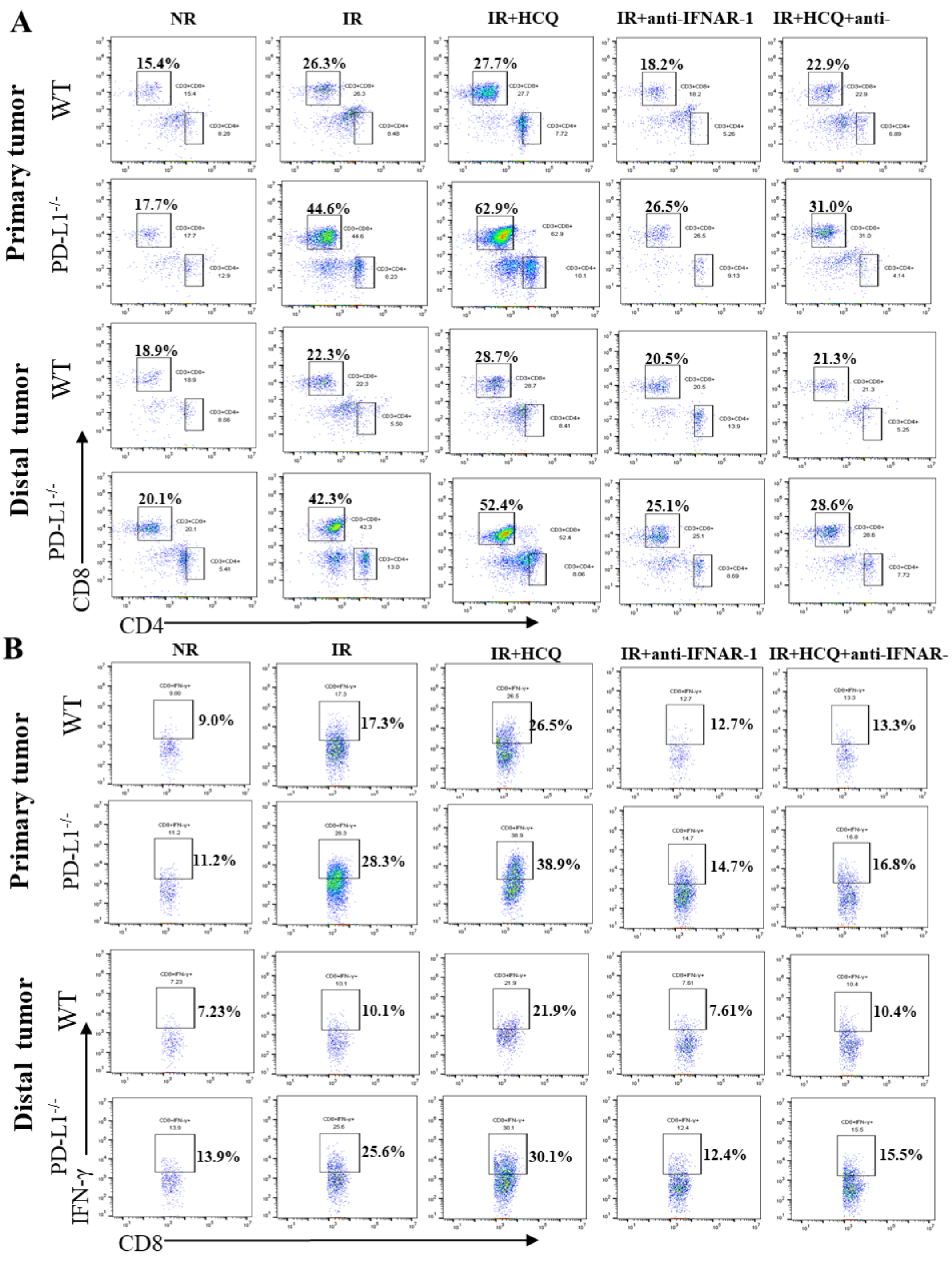

Figure S2. IR combined with PD-L1 deficiency and HCQ/anti-IFNAR1 enhanced the ratio and function of CD8+ T cells in tumors, related to Figure 7. Primary tumor in WT and PD-L1$^{-/-}$ mice was irradiated (IR) or non-irradiated (NR). HCQ (autophagy inhibitor) (50 mg/kg) was injected intraperitoneally 2 h before each IR, anti-IFNAR1 were injected intraperitoneally one day before first IR.

(A) Flow cytometeric assay of the percentage of CD8+T cells in primary and distal tumors of indicated groups. **(B)** Flow cytometeric assay of the percentage of IFN-γ positive cells within CD8+T cells in primary and distal tumors of indicated groups.

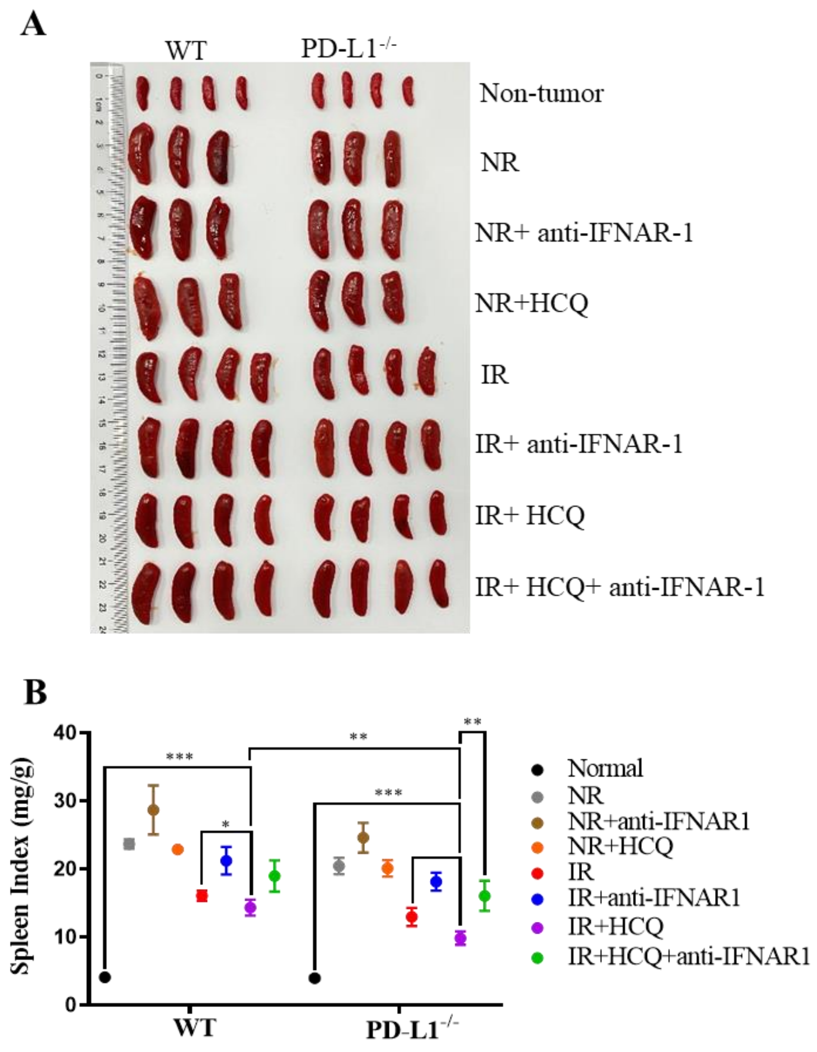

Figure S3. IR combined with PD-L1 deficiency and HCQ/anti-IFNAR1 inhibited the abnormal enlargement of spleen caused by tumor growth, related to Figure 7. **(A)** The photo of spleens in the WT and PD-L1$^{-/-}$ mice bearing LLC tumors. Primary tumor in WT and PD-L1$^{-/-}$ mice was irradiated (IR) or non-irradiated (NR). HCQ (autophagy inhibitor) (50 mg/kg) was injected intraperitoneally 2 h before each IR, anti-IFNAR1 were injected intraperitoneally one day before first IR. **(B)** The organ index of above spleen in mice.

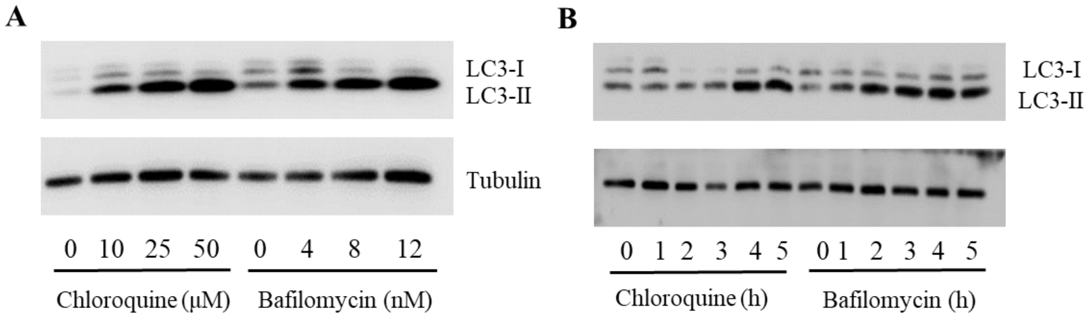

Figure S4. Effects of autophagy inhibitors of chloroquine (CQ) and bafilomycin with different concentrations and action times on LC3 protein expression in irradiated LLC cells, related to Figure 8.

(A) Western blot assay of LC3 expressions in LCC cells at 5 h after irradiation. The cells were pretreated with different concentrations of CQ (0, 10, 25, 50 μM) and bafilomycin (0, 4, 8, 12 nM) for 1 h before 4 Gy irradiation. **(B)** Western blot assay of LC3 expressions in LCC cells at different time point (0, 1, 2, 3, 4, 5 h) after irradiation under the pretreatment of 25 μM CQ and 8 nM bafilomycin for 1 h before 4 Gy irradiation.
